# The Efficacy of Rule-Based Versus Large Language Model-Based Chatbots in Alleviating Symptoms of Depression and Anxiety: Systematic Review and Meta-Analysis

**DOI:** 10.2196/78186

**Published:** 2025-12-04

**Authors:** Qiuxue Du, Yongliang Ren, Ze-long Meng, Han He, Shasha Meng

**Affiliations:** 1 Beijing Yuxin Technology Co., Ltd, Room 2-5, 13th Floor, Building 2, No. 48, Zhichun Road, Haidian District, Beijing, 100086, China, 86 010-81377053; 2 Department of Psychology, School of Humanities and Social Sciences, Beijing Forestry University, Beijing, China

**Keywords:** large language models, chatbots, mental health, depression, anxiety, PRISMA, Preferred Reporting Items for Systematic Reviews and Meta-Analyses

## Abstract

**Background:**

The global mental health crisis is becoming increasingly severe. Due to the shortage of mental health professionals, high treatment costs, and insufficient accessibility of services, there is an urgent need for scalable and low-cost intervention methods. In recent years, chatbots have shown potential for psychological interventions. The efficacy differences between large language model (LLM)-based and rule-based chatbots have not been systematically evaluated, with few studies directly comparing the two; existing meta-analyses have notable limitations: there is high heterogeneity in intervention design (eg, dialogue structure, interaction frequency, and duration) across studies, and there is a lack of direct comparison of differentiated intervention effects on depressive and anxiety symptoms, making it difficult to integrate conclusions.

**Objective:**

By integrating studies from the past five years, this research evaluates the differences in effectiveness between LLM-based and rule-based chatbots in alleviating depressive and anxiety symptoms. It also analyzes the impacts of control group type, intervention duration, and age on intervention outcomes. By analyzing chatbot functionality, the study aims to provide evidence-based technological pathway options and optimization recommendations for differentiated interventions for depression and anxiety.

**Methods:**

A systematic search of 7 databases included 15 studies published between 2020 and 2025. Robust variance estimation (RVE) was used to account for non-independent effect sizes, and standardized mean differences (SMDs) were calculated using Hedges *g*. Based on the expectation of clinical and methodological heterogeneity among studies, a random-effects model was preselected, and the pooled effect size was estimated using restricted maximum likelihood estimation (REML) and interpreted according to Cohen criteria. Publication bias was assessed using the RVE-adjusted Egger test, funnel plot asymmetry, and a fail-safe N.

**Results:**

For depression, rule-based intervention achieved a small but significant effect (*g*=0.266; 95% CI 0.020-0.512; *P*=.04), while LLM-based intervention showed a nonsignificant effect with wide confidence intervals (*g*=0.407; 95% CI −0.734 to 1.550; *P*=.17). For anxiety, rule-based intervention did not yield a significant effect (*g*=0.147; 95% CI −0.073 to 0.367; *P*=.15). Similarly, LLM-based intervention showed a higher point estimate but also with nonsignificance and wide confidence intervals (*g*=0.711; 95% CI −0.334 to 1.760; *P*=.13). Subgroup analysis showed that the rule-based chatbot was more effective than the blank control for depression, with the greatest effect in the medium term (4‐8 weeks).

**Conclusions:**

Rule-based chatbots have a modest effect on improving depressive symptoms and are suitable for environments with limited psychological resources; 4‐8 weeks may be a critical intervention window. Intervention duration and participant age did not significantly influence intervention effectiveness. Limited by the sample size, robust evidence supporting the effectiveness of LLM-based chatbot interventions is lacking, and further sample size expansion is warranted.

## Introduction

The global mental health crisis continues to intensify, with over 280 million people affected by depression and 301 million by anxiety disorders, contributing to annual economic losses exceeding US$1 trillion [1]. Over the past decade, the prevalence of depression and anxiety has shown a clear upward trend [2]. This rising prevalence underscores an urgent need for scalable and accessible interventions, especially given critical barriers such as a shortage of mental health professionals, high treatment costs, and structural inefficiencies [3]. Digital tools, particularly artificial intelligence (AI) chatbots, have emerged as promising solutions due to their affordability, anonymity, and accessibility [4].

At present, there are 2 main pathways for the application of chatbots in the field of mental health. The first pathway involves rule-based intervention frameworks, which use structured treatment frameworks or intent recognition to dynamically constrain the output content. This method has advantages in ensuring intervention effectiveness, but its interaction mode is seminatural language, which limits semantic understanding and flexibility, often hindering the formation of a therapeutic alliance [5]. In the past 5 years, with the advancement of deep learning technologies, a second pathway has emerged: intervention frameworks based on large language models (LLMs). By analyzing vast amounts of user data and conversation records, these models can simulate more complex and natural human dialogue patterns and provide personalized responses based on real-time feedback [6]. However, this openness also presents significant challenges, including the uncontrollable nature of generated content (“hallucinations”), the opacity of decision-making processes (“black box” issues), and data privacy concerns [7].

Most current meta-analyses fail to distinguish between different technology approaches, typically including chatbots based on various AI technologies. These intervention technologies exhibit significant differences in interaction depth, semantic understanding, and emotional support capabilities. This not only leads to high heterogeneity in meta-analytic results but also fails to reveal the unique contributions of specific AI technologies in psychological interventions, and even more so, it is difficult to determine the specific impacts of different AI technology approaches in mental health interventions [8,9]. Simultaneously, existing research primarily focuses on the technical performance and basic applications of chatbots, while there is still a lack of in-depth exploration of their effectiveness and applicability in real psychological health scenarios [10]. Moreover, the vast majority of studies remain at the level of evaluating the overall intervention effects, lacking systematic dissection and analysis of the internal functional components of chatbots. This makes it difficult to identify the core factors that are truly effective and also limits the precise design and optimization of intervention tools. Therefore, independent research on LLM is necessary to clarify its actual effectiveness in mental health settings and compare the advantages and disadvantages of the 2 technologies. This will provide empirical evidence for the selection and design of mental health intervention tools and promote the development of precise and scalable solutions.

Through systematic review and meta-analysis, this study focuses on the application of LLM-based chatbots in psychological intervention and compares them with traditional rule-based intervention methods. Specific research questions include (1) comparison of the effectiveness of the 2 intervention techniques in improving depression and anxiety symptoms; (2) analysis of heterogeneity of effects across different subgroups (control group type, age, and intervention duration); and (3) conduct component-level analysis of chatbots, analyze the strengths and weaknesses of different components, and explore feasible optimization solutions.

## Methods

### Overview

This study has been preregistered on the PROSPERO (International Prospective Register of Systematic Reviews) platform (Registration Number: CRD420250653433). During the implementation process, we optimized the search strategy, inclusion and exclusion criteria of the original registration scheme, and added a rule-based chatbot intervention. To reduce the risk of regional bias and enhance the comprehensiveness of the search, we added the China National Knowledge Infrastructure (CNKI) and Wanfang Chinese databases and expanded the keywords related to LLMs. We clearly defined “adult” as individuals older than 14 years and excluded studies involving children, cognitively impaired patients, and those that did not report or could not calculate effect sizes, ensuring consistency among the included studies.

### Literature Search

Based on the PRISMA (Preferred Reporting Items for Systematic Reviews and Meta-Analyses; Checklist 1) flow diagram, the target literature was retrieved using the following 3 methods.

English literature was primarily searched in the PubMed, Cochrane Library, Scopus, Embase, and PsycINFO databases using combinations of keywords such as “Generative Artificial Intelligence,” “Machine Learning,” “Chatbot,” “Distance Counseling,” “Online Psychotherapy,” “Mental Health,” and “Personal Satisfaction.” Chinese literature was searched through CNKI and Wanfang databases using combinations of keywords, such as “Artificial Intelligence,” “Chatbot,” “Mental Health,” and “Digital Therapy.” The complete search strategy is provided in Multimedia Appendix 1.

We reviewed relevant systematic reviews and meta-analyses on the topic and used the reverse snowball search strategy. This involves examining the reference lists of these pertinent studies as well as identifying studies that have cited these key papers to retrieve additional literature that may have been missed in the initial keyword search.

Finally, to avoid omissions, we manually searched the top journals in the target field and continuously tracked the latest published related literature. Although the study did not conduct a systematic search of gray literature, we supplemented this with manual searches of clinical trial registries and websites of relevant academic institutions to minimize publication bias [11].

The search timeframe spans from January 2020 to July 2025. The selection of this window is grounded in concrete real-world contexts and technological development considerations. First, the global outbreak and ongoing impact of the COVID-19 pandemic have significantly reshaped the delivery of mental health services, leading to an exponential increase in the demand for online and remote psychological services post-2020 [12]. This dramatic shift not only accelerated the practice of remote psychological service models but also directly stimulated a surge in related research literature, providing a highly relevant and rich analytical foundation for this study [13]. Second, before 2020, natural language processing (NLP) technologies were not fully mature, with many applications still in the exploratory phase and unable to offer natural and comprehensive mental health services [14]. Since 2020, advancements in computational power and the availability of large-scale datasets have spurred rapid development in LLMs, providing innovative tools and methods for fields such as NLP, affective computing, and dialogue systems, and demonstrating vast application prospects [15]. Thus, restricting the search scope to 2020‐2025 allows us to precisely focus on the transformation in service models and the surge in research triggered by the pandemic, while concurrently capturing the initial application of LLMs and their systematically explored, validated, and discussed potential in the mental health field. The last search was conducted on July 21, 2025.

### Inclusion and Exclusion Criteria

Inclusion and exclusion criteria were based on the population, intervention, comparator, outcome, timing, and study design (PICOTS) principle [16].

P (Population): the study included individuals aged 14 years or older, both healthy and those experiencing psychological distress, as well as participants with clinical or subclinical diagnoses of depression or anxiety. Excluded were children under 14 years (due to guardian consent and ethical restrictions) [17], individuals at risk of suicide, those with severe cognitive impairments or psychotic disorders (such as schizophrenia and bipolar disorder), and participants with neurological conditions affecting the study (like dementia and brain injury). Participants were categorized by age into young (<30 years), middle-aged (30‐50 years), and old (>50 years) groups, reflecting different life cycle stages [18].

I (Intervention): this study focused on 2 types of chatbot interventions, LLM-based chatbots must use a LLM for text comprehension and generation and support simultaneous 2-way interaction with users. Rule-based chatbots are defined as those that follow specific rules, decision trees, or fixed options [19]. They achieve limited interactive responses through predefined intent recognition, keyword matching, or streamlined dialogue paths. Their response logic strictly adheres to preprogrammed algorithmic rules and lacks the ability to autonomously generate content. Both types of interventions must meet the criteria for independent implementation, excluding research designs that combine them with other psychological interventions (such as mindfulness training and bibliotherapy) to ensure clear attribution of intervention effects.

C (Comparison): the control groups are categorized into 3 types based on the nature of the intervention, blank control (blank), bibliotherapy (book), and standard human care (human). The human group refers to various psychological interventions provided by counselors, doctors, or nurses. The book group involves psychological regulation implemented by participants themselves through standardized psychology self-help books or structured reading materials. The blank group receives no active intervention, only regular observation or waitlist control. Studies with designs that cannot clearly distinguish between control measures and experimental interventions are excluded to ensure the validity of intergroup comparisons.

O (Outcome): must report at least one mental health outcome related to depression or anxiety. Studies that did not report any of these mental health indicators were excluded.

T (Time): included studies must clearly report the duration of the intervention. This study categorizes the intervention duration into short-term (<4 weeks), medium-term (4‐8 weeks), and long-term (≥8 weeks). Studies that do not clearly specify the intervention duration will be excluded. Furthermore, studies with intervention durations exceeding 26 weeks will not be included, as longer durations are not suitable for evaluating the effects of interventions. These long durations are prone to interference from factors unrelated to the intervention, such as user engagement, AI technology iteration, resource costs, and external environmental influences.

S (Study design): randomized controlled trials (RCTs) and quasiexperimental designs were included, and comparative data between the experimental and control groups were required; studies in both English and Chinese are accepted. Must report data relevant to meta-analysis, such as means, SDs, and effect sizes (eg, Cohen *d* and Hedges *g*), or provide sufficient information to calculate effect sizes if not directly reported. Studies with significant baseline differences between groups or those that did not report clear outcome measures were excluded.

Through a systematic selection process, 15 studies that met the inclusion criteria were ultimately included. For studies containing multiple independent experiments (such as different intervention conditions or measurement time points), effect size data were extracted separately, resulting in a total of 40 effect sizes. An RVE method was used for the meta-analysis to address the dependency among effect sizes.

### Literature Screening Process

Literature screening includes 2 stages, initial screening of titles and abstracts and fine screening of full texts.

The initial screening is divided into 2 parts, AI batch analysis and manual review. First, Zotero (Corporation for Digital Scholarship) literature management software is used to remove duplicates and construct prompt words. GPT (OpenAI) and DeepSeek (DeepSeek AI) tools are used to batch analyze literature titles and abstracts to exclude literature that does not meet the requirements. Subsequently, authors QD and HH of this article manually reviewed the abstracts of the literature screened by AI. The 2 independently completed the evaluation and cross-checked the results. If there are differences of opinion, the 2 will discuss and try to reach a consensus. If there is still a dispute, the third researcher, ZM, will arbitrate and make the final decision. The selected literature enters the full text fine screening stage.

In the full text fine screening stage, QD and HH independently read and evaluate the full text according to the pre-established PICOTS inclusion and exclusion criteria and make a preliminary judgment on whether to include it. Afterward, the 2 cross-checked their respective decisions. For the literature with disagreements, QD and HH discussed again to seek consensus; if no consensus could be reached, author ZM made the final decision. The final included literature will be used for meta-analysis to ensure the comprehensiveness and reliability of the study.

### Data Extraction and Quality Assessment

Microsoft Excel 2016 was used to extract and evaluate, including 3 stages developing a data coding table, independent coding, and a quality assessment table.

The data coding table, developed based on previous AI or chatbot-related literature, was created by the first 3 authors and is provided in Multimedia Appendix 2 [20]. The extracted data features include: (1) publication characteristics author, year, and country; (2) intervention characteristics intervention type (RCT and non-RCT), intervention content (LLM-based and rule-based), and intervention duration (short, medium, and long); (3) control characteristics control measures (human, book, and blank); (4) participant characteristics participant age (young, middle, and old), participant gender, number of participants, dropout rate; and (5) outcome measures psychological traits (depression and anxiety), measurement scales (standardized tools for quantifying psychological traits).

In the independent coding stage, the 2 authors QD and HH extracted data from the literature included in the meta-analysis according to the coding table. When coding, the results reported based on the intention-to-treat (ITT) principle were preferred. If the study only reported the per-protocol (PP) analysis, the results were collected and recorded and explained. At the same time, since the effect size must be reported or the statistical value of the calculable effect size must be provided when the literature is included, there is no missing effect size. For minor missing of individual noncore variables, such as demographic information, since it does not affect the core research questions, the status quo is retained and no interpolation or estimation is performed at the meta-analysis level. The 2 authors QD and HH extracted data from the literature included in the meta-analysis according to the codebook, and any disagreements were arbitrated by author ZM to reach a final consensus.

The quality of the RCTs and non-RCTs included in the meta-analysis was assessed using the RoB 2.0 tool (Risk of Bias 2.0; Cochrane Collaboration) and the JBI tool (Joanna Briggs Institute) for assessing bias in nonrandomized intervention studies, respectively, according to the Cochrane Handbook and CONSORT-AI (Consolidated Standards of Reporting Trials–Artificial Intelligence) guidelines [21,22]. This assessment was independently performed by authors QD and HH, who cross-checked the results. In case of discrepancies in the scores, the original manuscripts were reread and the authors YR and ZM were invited to participate in the discussion until consensus was reached.

### Effect Size Calculation

This study used R 4.5.1 (R Core Team) in combination with robumeta, metafor, and other packages for meta-analysis. The RVE method was used to handle dependent effect sizes, and the calculation and conversion of effect sizes were completed using CMA 3.0 software (Comprehensive Meta-Analysis version 3.0; Biostat, Inc) [23].

All effect sizes were calculated based on the SMD using Hedges *g* to eliminate differences in measurement tools and sample sizes across studies. The direction of the effect size was uniformly assumed to be positive, indicating greater symptom improvement in the intervention group. Included studies were required to directly report effect sizes or provide complete raw data for effect size calculation, ensuring that no effect size could be calculated due to missing data. The size of the combined effect size was interpreted according to Cohen criteria, with a small effect size of 0.2, a medium effect size of 0.5, and a large effect size of 0.8 [24].

For nonindependent effect sizes in included studies (such as multiple outcome measures, multiple time-point measurements, or multiple experimental group comparisons), direct application of traditional meta-analytic methods (assuming independence of effect sizes) can lead to underestimated SEs and overly narrow confidence intervals. Therefore, each study was treated as a cluster, and the dependency structure was addressed using the correlation weighting method within the RVE framework [25]. Furthermore, based on correlation reports from previous similar literature, the within-study effect size correlation coefficient was set to ρ=0.8 [26]. Quasi-robust SEs and adjusted *t* tests (2-tailed) were used to control for bias, and the Kenward-Roger small sample correction was applied to enhance estimation precision. The final model was used to calculate the pooled effect size and its 95% CI using restricted maximum likelihood estimation (REML).

### Heterogeneity Analysis and Publication Bias

Due to expected heterogeneity among included studies in intervention design, sample characteristics, and measurement tools, this study indirectly assessed the degree of variability using model-based adjusted SEs and confidence intervals within the RVE framework, rather than relying on traditional heterogeneity measures (such as *I*²) to enhance the explanatory power and credibility of the results [27]. Heterogeneity was assessed using the between-study variance τ², following the Higgins criteria: <0.04 is considered low heterogeneity, 0.04‐0.16 is considered moderate, and >0.16 is considered high [28]. To clarify the sources of heterogeneity, we first tested the moderating effect of categorical variables through subgroup analyses and assessed the significance of between-group differences using the Wald test. Meta-regression analyses were then conducted for continuous variables, with slope estimates and their 95% CIs calculated.

Publication bias refers to the phenomenon in which statistically significant findings are more likely to be published, leading to biased effect sizes. This bias can affect the reliability of systematic reviews and meta-analyses, potentially exaggerating intervention effects or underestimating adverse outcomes. It is an objective and difficult-to-avoid problem [29]. First, asymmetry was visually checked using a funnel plot of the RVE-corrected SE. Next, an RVE-adjusted Egger test was performed using the *weightr* package. An intercept *P*<.10 indicated possible publication bias. Finally, a fail-safe N was calculated. Results were considered robust when the number of ineffective studies required to be included exceeded “5k+10” (k being the original number of studies). These 3 conclusions were mutually reinforcing, ensuring the reliability of the bias assessment [30].

## Results

### Literature Inclusion

**Figure 1.**
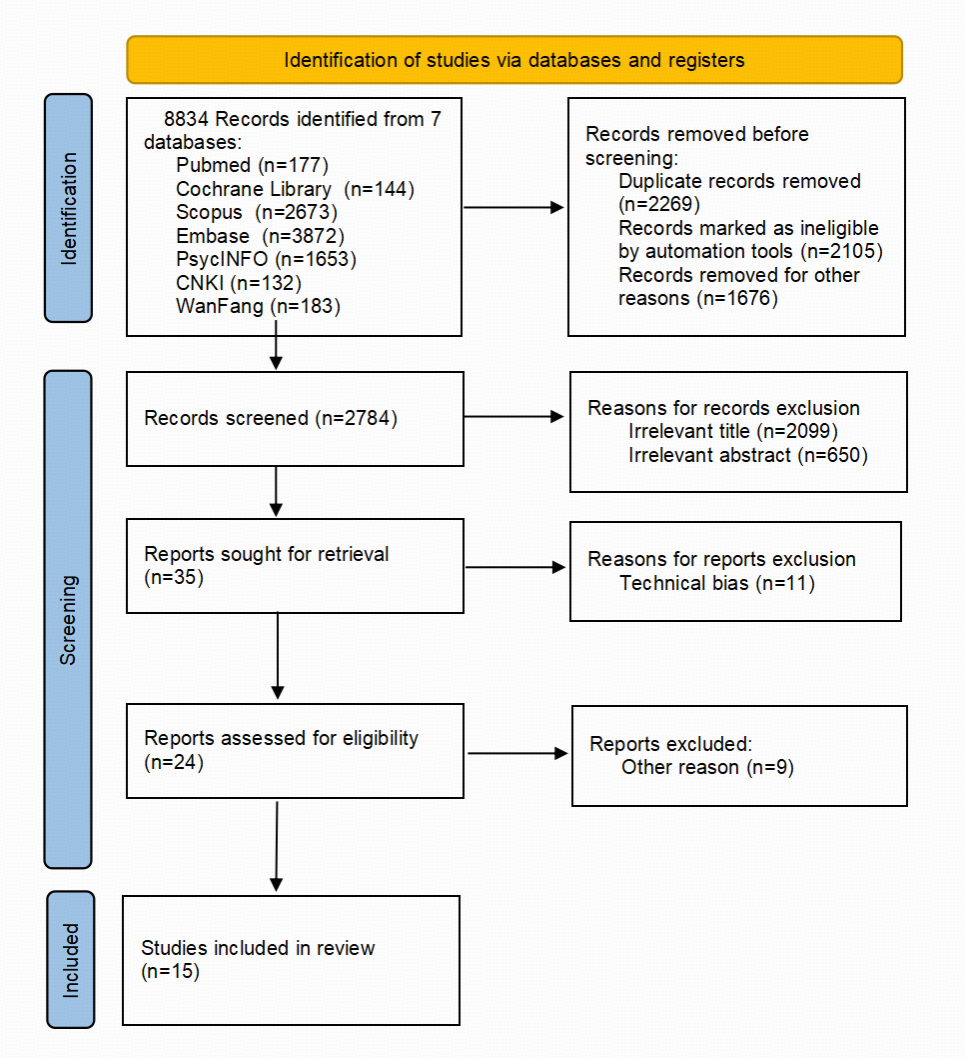
Literature screening flowchart.

Literature screening consisted of 3 stages: search, screening, and inclusion. Two researchers independently screened and evaluated studies according to the inclusion and exclusion criteria. In case of uncertainty, the third author or corresponding author made the final decision. As shown in Figure 1, 8834 articles were identified from the database after the initial screening, including 315 Chinese articles and 8519 English articles. By constructing prompt words and integrating Zotero literature management software with AI tools such as GPT and DeepSeek, batch analysis of titles and abstracts was performed. After removing duplicates and articles with low relevance to the study, 2784 articles remained. These titles and abstracts were manually reviewed, and a total of 35 articles entered the full-text screening stage. These articles were independently evaluated by two researchers and screened in detail according to the PICOTS principles. Fifteen articles were ultimately included in the meta-analysis, of which 13 were RCTs and 2 were quasiexperimental designs.

### Description of Included Studies

The basic information of the literature is summarized in (Multimedia Appendix 2). A total of 15 articles were included, of which 9 were chatbots that intervened based on rules and 6 were chatbots that intervened based on LLM.

Existing literature shows a clear trend of regional concentration among research participants, with the United States and China accounting for the highest proportion (67% combined). Publication dates were primarily between 2023 and 2025, reflecting the intense interest in AI research. In terms of experimental design, intervention periods were generally short, typically ranging from one week to one month. The average dropout rate was 15%, indicating reasonable compliance [31]. Sample characteristics revealed an average age of approximately 38 years, with a slightly higher proportion of women than men (57% vs 43%). Recruitment was primarily through online channels such as social media, consistent with the trend toward digital research. Control group interventions were categorized into 3 types, human intervention involving counselors and nurses (human), bibliotherapy with self-help books or learning materials (book), and a blank control (blank). The experimental groups included both rule-based and LLM-based chatbots.

Among the 15 studies [32-46] included in the meta-analysis, there were 13 RCTs [32,34,36-46]. The meta-analysis incorporated all eligible RCTs from previous meta-analyses, as well as newly published studies from 2025 onwards. Nine studies [34,37,38,40-42,44-46] assessed both depression and anxiety symptoms, whereas 6 [32,33,35,36,39,43] focused solely on either depression or anxiety.

### Heterogeneity Testing

**Figure 2.**
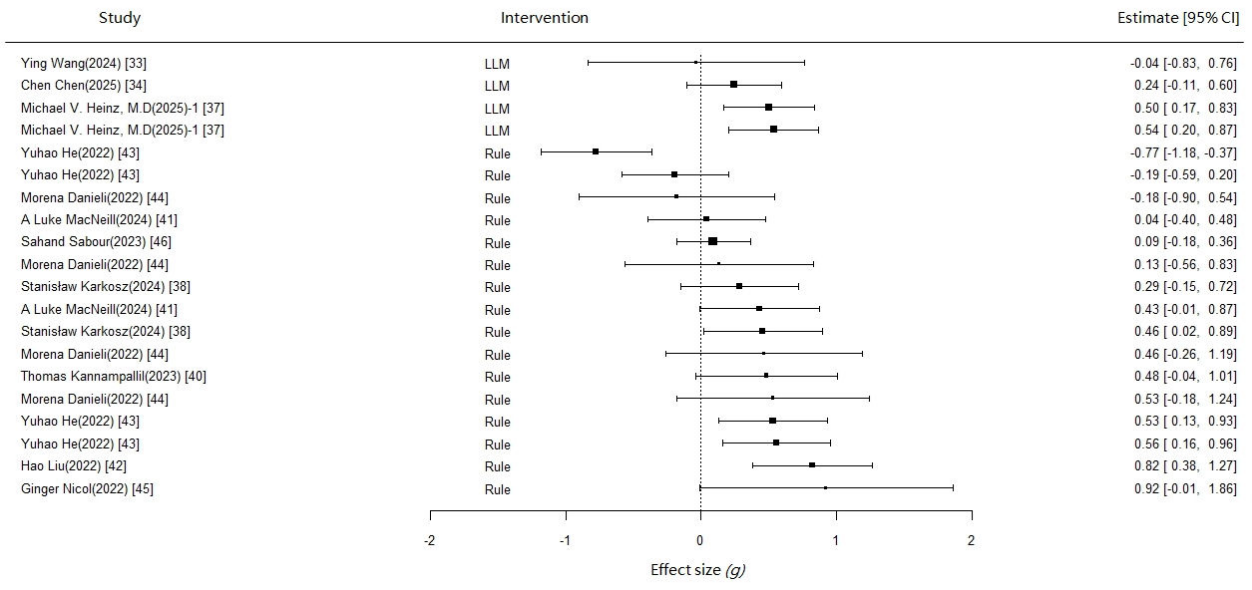
Depression forest plot [33,34,37,38,40-46].

This study used the RVE method to address issues of variance heterogeneity and correlation among effect sizes. The degree of heterogeneity was comprehensively evaluated using between-study variance (τ²) and the RVE-adjusted *I*² statistic (see Table 1). The standard reference values for heterogeneity are: τ²<0.04 indicates low heterogeneity, 0.04‐0.16 indicates moderate heterogeneity, and >0.16 indicates high heterogeneity [47]; RVE-adjusted *I*² uses thresholds of 20% and 75% to distinguish between low, moderate, and high heterogeneity. Forest plots were also drawn to assist in visual evaluation (Figures2  3).

For the intervention effect analysis on depressive symptoms, LLM-based chatbots demonstrated complete homogeneity (τ²=0; *I*²=0%), although this result is based on only 3 studies, which may affect the reliability of the estimates. Rule-based chatbots also showed ideal homogeneity (τ²=0; *I*²=0%), but the within-study heterogeneity was relatively high (ω²=0.173), suggesting the need to consider the dependency among multiple effect sizes within the same study; further analysis could be conducted using meta-regression.

In the intervention for anxiety symptoms, LLM-based chatbots showed high heterogeneity (τ²=0.321, *I*²=83.7%), indicating substantial differences might exist in different LLM intervention schemes or population characteristics, warranting subgroup analysis or meta-regression to explore the sources of heterogeneity further. However, the heterogeneity for rule-based chatbots was within an acceptable range (τ²=0.034, *I*²=28.6%), and potential moderating variables could be examined in future research to enhance the robustness of the conclusions.

**Table 1.** Results of heterogeneity testing.

Outcome and type		Number of studies (Effect sizes)	Tau²^a^ (τ²)	Omega²^b^ (ω²)	RVE^c^-adjusted *I*²	Effect size (*g*)^d^, (95% CI)
Depression						
LLM^e^		3 (4)	—^f^	—	0.0	0.407 (−0.734 to 1.550)
Rule^g^		8 (16)	0.0	0.173	0.0	0.266 (0.020 to 0.512)
Anxiety						
LLM		5 (7)	0.321	0.0	83.7	0.711 (−0.334 to 1.760)
Rule		8 (13)	0.034	0.0	28.6	0.147 (−0.073 to 0.367)

a Tau²: between-study heterogeneity, the degree to which the true effect size varies between studies.

b Omega²: within-study heterogeneity, the degree of variability between effect sizes within the same study.

c RVE-adjusted I²: the relative proportion of total heterogeneity contributed by between-study heterogeneity (Tau²).

d Effect Size (*g*): Standardized effect size, Hedges *g*.

e LLM: large language model.

f Not applicable (fewer than 3 studies included).

g Rule: rule-based chatbot.

**Figure 3.**
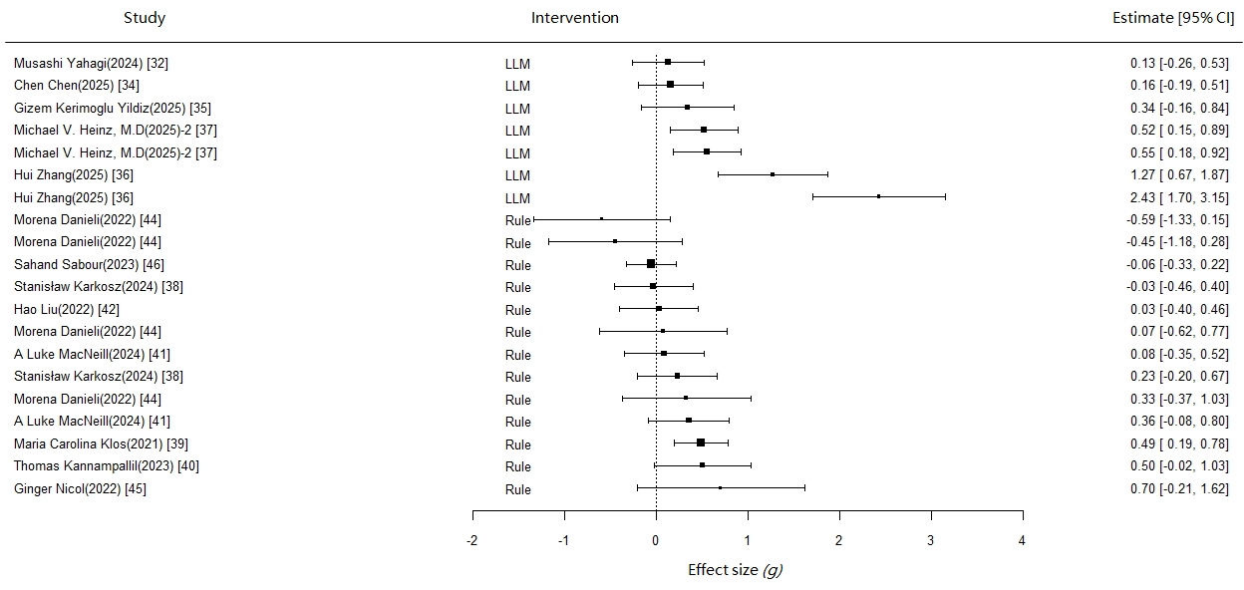
Anxiety forest plot [32,34-42,44-46].

### Quality Assessment Results

**Figure 4.**
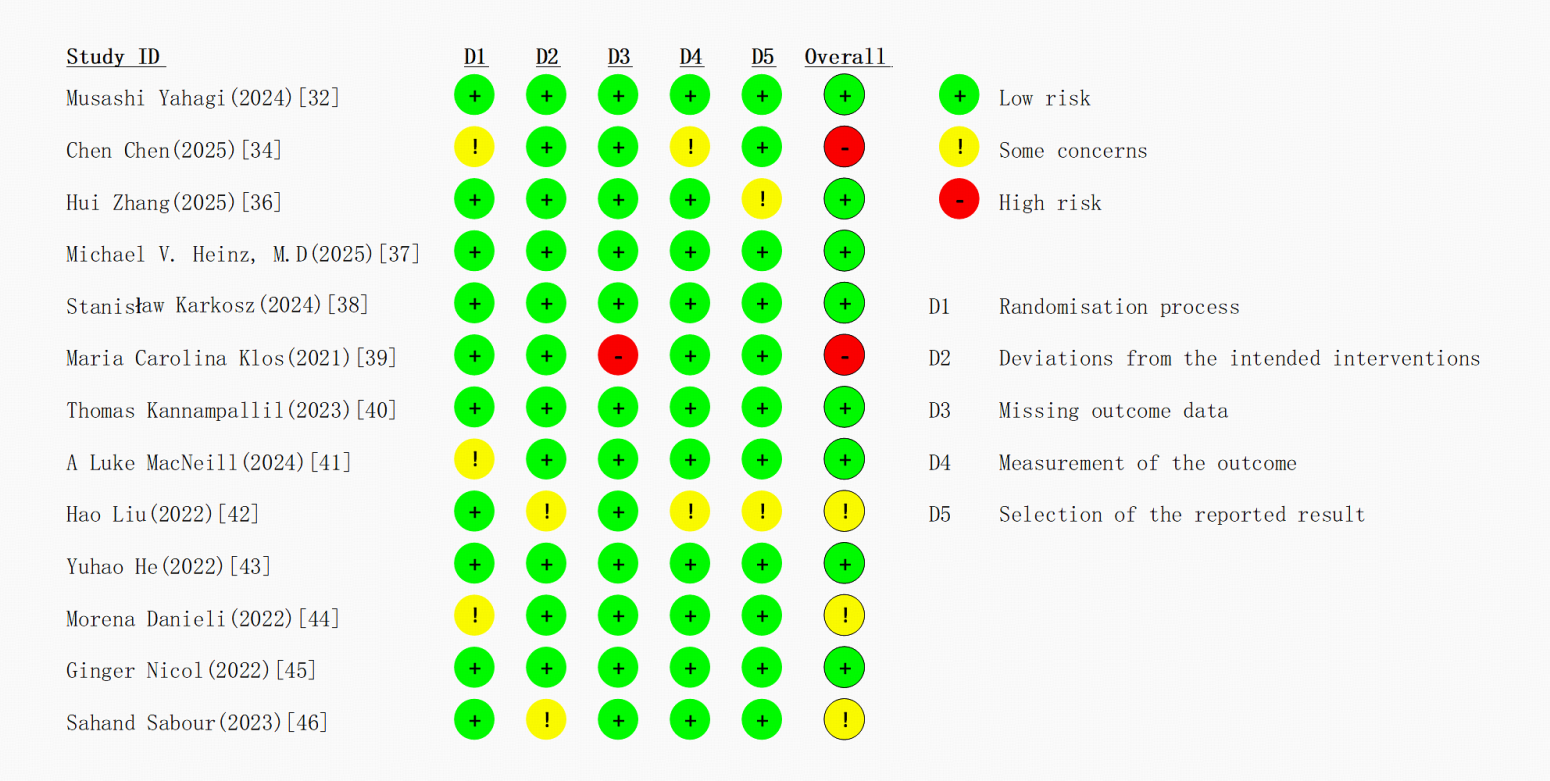
Quality assessment results [32,34,36-46].

The methodological quality of the 13 included RCTs [32,34,36-46] was assessed using the Cochrane RoB 2 tool (Figure 4). In terms of the randomization process, 10 studies explicitly reported the generation and concealment of the random allocation sequence and were rated as low risk. The remaining 3 studies were assessed as having some risk due to inadequate description of the allocation concealment. The adherence to interventions showed that none of the studies significantly deviated from the intended intervention protocols. Among these, 3 studies [32,34,36], where interventions were directly implemented by health care personnel, demonstrated exceptionally high adherence. The remaining studies effectively maintained participant completion rates through regular email reminders and follow-up monitoring. The remaining 2 quasiexperimental studies were evaluated using the JBI tool and were both found to be of moderate to high quality.

Regarding the completeness of outcome data, one study was judged to be at high risk due to a high dropout rate (72%), while the missing data rate in the other studies was around 10%. These were assessed as low risk, as they were confirmed to be missing completely at random through Little’s MCAR test [48]. All studies used validated standardized scales (eg, Patient Health Questionnaire–9, PHQ-9; State–Trait Anxiety Inventory, STAI) for outcome measurement, with good reliability and validity, indicating a low risk of measurement bias. The assessment of selective reporting indicated that despite the presence of multiple measurement time points, all studies comprehensively reported the predefined primary outcome measures.

The average dropout rate for all studies included in this study was 15%, which is consistent with the overall dropout rate in psychotherapy over the past 20 years (19.7%) [49]. This rate is comparable to the dropout rates for psychological interventions using conversational agents (24%) and mindfulness applications (21.84%) but lower than the dropout rate for smartphone-based interventions (50%) [50].

### Effect Size Analysis

This study used the RVE method to handle nonindependent effect sizes, with studies clustered and an intraclass correlation of ρ=0.8. Hedges *g* standardized mean differences were aggregated using REML [51], with effect sizes interpreted according to Cohen standards. All analyses were conducted using R (version 4.5.1; R Core Team), specifically using the *robumeta* and *metafor* packages. The Kenward-Roger correction was applied to ensure the accuracy of estimates with small samples.

The overall meta-analysis indicates that chatbot interventions produce a small but statistically significant improvement in depressive symptoms (*g*=0.283; 95% CI 0.086-0.48; *P*=.01). In contrast, there was no significant improvement in anxiety symptoms (*g*=0.333; 95% CI −0.025 to 0.690; *P*=.07). However, the 95% CI crosses zero, and the *P* value is at a marginal level of significance, suggesting these results should be interpreted with caution. Additionally, due to significant clinical heterogeneity among the studies, the combined results warrant careful interpretation.

Further subgroup analysis revealed differentiated effect patterns of intervention types on depressive and anxiety symptoms. For depressive symptoms, rule-based interventions showed a significant small effect size improvement (*g*=0.266; 95% CI 0.020-0.512; *P*=.04). However, the effect of LLM-based interventions did not reach statistical significance (*g*=0.407; 95% CI −0.734 to 1.550; *P*=.17), which may be limited by sample size or heterogeneity. For anxiety symptoms, neither of the 2 interventions showed significant improvement effects. The effect of rule-based chatbot interventions was small and nonsignificant (*g*=0.147; 95% CI −0.073 to 0.367; *P*=.15). Similarly, due to the limited number of studies on LLM-based chatbots, the intervention effect was not significant and had a wide confidence interval, indicating substantial uncertainty in the results (*g*=0.711; 95% CI −0.334 to 1.760; *P*=.13).

To directly compare the effects of the 2 intervention types, we conducted an intergroup effect size comparison. The results showed that for improving depressive symptoms, the effect size of rule-based interventions was 0.095 lower than that of LLM-based interventions (95% CI −0.754 to 0.565). For improving anxiety symptoms, the effect size for rule-based interventions was 0.525 lower than for LLM-based interventions (95% CI −1.311 to 0.26), though neither reached statistical significance. This suggests that although LLM interventions tend to show a larger effect size in both depressive and anxiety symptoms, the difference in effects between these two types of interventions is not practically significant. Notably, the comparison is based on 9 rule-based intervention studies and 6 LLM-based studies, and this imbalance in the number of studies may affect the robustness of the comparison. Future research could explore their applicable contexts and optimization directions further, considering specific intervention mechanisms and patient characteristics.

### Publication Bias

This study systematically assessed publication bias using three methods: the funnel plot, RVE-adjusted Egger test, and the fail-safe N analysis. The results indicate that the impact of publication bias on the estimation of the overall effect is negligible, thus supporting the high credibility of the study’s conclusions.

**Figure 5.**
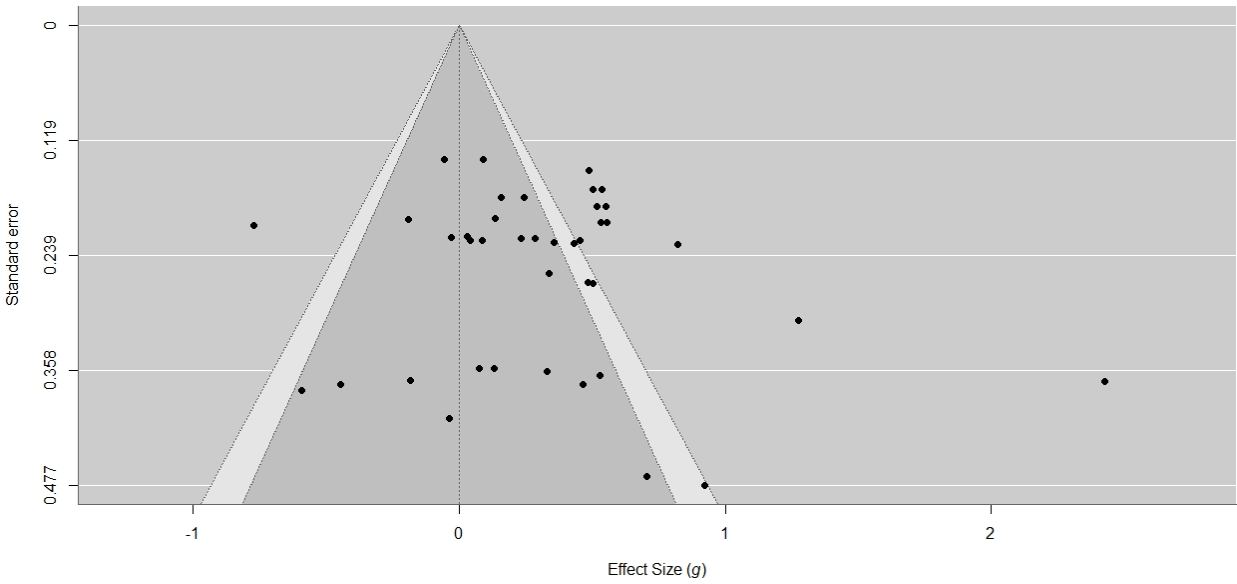
Funnel plot.

The funnel plot reveals a pattern of centrally clustered and generally symmetrical distribution of effect size points. Although there is slight asymmetry on both sides, there is no significant indication of publication bias (see Figure 5). To further validate this result, we used the RVE-based Egger regression test, using the CR2 adjustment in the *clubSandwich* package to control for the nonindependence of effect sizes. The regression results showed no significant association between SE and effect size (*β*=1.59; SE=1.74; *t*_4.85_=0.92; *P*=.40), and the intercept was also not statistically significant (*β*=−.008; SE=0.34; *t*_7.66_=−0.02; *P*=.98). These findings further exclude the possibility of publication bias. Finally, the Rosenthal fail-safe N analysis suggests that as many as 920 unpublished zero-effect studies would be required to overturn the current significant research conclusions (*P*<.001). This number far exceeds the conventional threshold (5k+10=85), providing strong support for the robustness of the study’s findings.

### Subgroup Analysis

This study primarily focuses on two types of chatbots and analyzes their differential effects on depression and anxiety interventions compared to various control measures (human, book, and blank), intervention durations (short, medium, and long), and participant ages (young, middle, and old). Subgroup analyses and intergroup comparison methods were used to conduct the study. To minimize bias due to an insufficient number of studies within subgroups, only subgroup analysis results with at least 3 studies per group were included. Furthermore, RVE meta-regression (CR2 correction) was used for extended analysis of continuous variables, specifically age and intervention duration.

#### Control Group Types

A robust variance estimation model (ROBUST [Randomized, Open-Label, Blinded Endpoint Study]) was used for the meta-analysis, directly estimating the effect size differences between the intervention and control groups through a no-intercept model. An intraclass correlation coefficient ρ=0.8 was set to control for interstudy correlation, with results presented in Table 2. To visually present the effect sizes and their confidence intervals, we generated forest plots for subgroups with more than 3 studies (see Multimedia Appendix 3).

In the analysis of depression intervention effects, rule-based chatbots demonstrated a statistically significant but small effect over the blank control group (*g*=0.288; *P*=.047). However, no significant difference was found when they were compared to bibliotherapy (*g*=0.237; *P*=.40). These results suggest that while rule-based chatbots may constitute a viable intervention option when resources are limited, they are not superior to existing, established interventions. Furthermore, because the number of included studies for LLM-based chatbots and other subgroups was fewer than 3, the analyses for these groups were not sufficiently robust to draw definitive conclusions.

In the analysis of anxiety intervention effects, rule-based chatbots did not show a statistically significant difference compared to either the blank control group (*g*=0.086; *P*=.63) or bibliotherapy (*g*=0.206; *P*=.27). Based on only 3 studies [32,34,36], LLM chatbots also showed nonsignificant difference compared to human intervention (*g*=0.951; *P*=.31), with notably wide confidence intervals. More research is urgently needed to robustly assess their efficacy. Other subgroups were not further analyzed due to fewer than 3 studies.

**Table 2.** Subgroup analysis of control group types.

Outcome, type^a^, and control			*g* ^b^	SE	*P* value	95% CI
Depression						
Rule^c^						
Human			0.330	—^d^	—	—
Book			0.237	0.209	.40	−0.978 to 1.451
Blank			0.288	0.094	.047	0.006 to 0.570
Anxiety						
LLM^e^						
Human			0.951	0.679	.31	−2.22 to 4.12
Book			0.339	—	—	—
Blank			0.536	—	—	—
Rule						
Human			0.201	—	—	—
Book			0.206	0.133	.27	−0.417 to 0.828
Blank			0.086	0.161	.63	−0.419 to 0.592

a Type: chatbot intervention type.

b *g*: Hedges *g*.

c Rule: rule-based chatbot.

d Not applicable (fewer than 3 studies included).

e LLM: large language model.

#### Intervention Duration

This study systematically examined the impact of intervention duration on the effectiveness of chatbots in psychological interventions through multidimensional analytical methods, revealing the dynamic characteristics of time effects and their potential mechanisms. First, subgroup analyses were conducted on 3 types of intervention durations (see Table 3). Second, an intercept-inclusive linear model was constructed for intergroup comparisons (see Table 4). Finally, a meta-regression analysis on the continuous variable of intervention days was performed.

In terms of improving depressive symptoms, a relatively clear temporal pattern was observed for rule-based interventions, whereas data for LLM-based interventions remained inconclusive due to an insufficient number of studies. For rule-based chatbots, the effect size demonstrated a steady increase with longer intervention duration: short-term (*g*=0.035), medium-term (*g*=0.383), and long-term (*g*=0.438). Although the medium-term effect did not reach conventional statistical significance (*P*=.10), the trend suggested that 4‐8 weeks might be a potential critical window for clinical improvement. Using short-term intervention as the reference group, further intergroup comparison analysis also indicated that rule-based interventions showed significant advantages at medium-term (*β*=.340; *P*=.04).

Regarding the intervention effects on anxiety symptoms, both types of chatbots also exhibited different temporal dynamics. LLM-based interventions have a larger but not significant effect in the short term (*g*=0.817; *P*=.24). Similarly, rule-based interventions were not significantly effective in improving anxiety in the short (*g*=−0.019; *P*=.69), medium (*g*=0.335; *P*=.09), or long term (*g*=0.124; *P*=.59). Comparison analysis at different time points indicated that rule-based interventions performed significantly better in the medium term relative to the short term (*β*=.359; *P*=.05). This pattern again suggests that the medium term (4‐8 weeks) may be a potential window for intervention, but the absolute efficacy during this period requires further confirmation.

Notably, meta-regression analysis showed that the effect of the number of intervention days on effect size was not significant, suggesting that within the scope of this study, intervention duration might not be a key factor influencing intervention efficacy.

**Table 3.** Subgroup analysis of intervention time.

Outcome and type^a^		Short-term^b^ (*g*^c^, *P*, k^d^, 95% CI)	Medium-term^e^ (*g*, *P*, k, 95% CI)	Long-term,^f^ *g*, *P*, k, (95% CI)
Depressive				
LLM^g^		0.245, —^h^, 1, —	0.501, —, 1, —	0.37, —, 2, —
Rule^i^		0.035, .75, 4, (−0.301 to 0.371)	0.383, .10, 4, (−0.143 to 0.909)	0.438, .17, 3, (−0.547 to 1.420)
Anxiety				
LLM		0.817, .24, 4, (−1 to 2.64)	0.552, —, 1 —	0.519, —, 1, —
Rule		−0.019, .69, 3 (−0.225 to 0.187)	0.335, .09, 3 (−0.155 to 0.825)	0.124, .59, 4 (−0.598 to 0.845)

a Type: Chatbot intervention type.

b Short-term: intervention duration＜ 4 weeks.

c *g*: Hedges *g*.

d k: number of studies.

e Medium-term: Intervention duration 4-8 weeks.

f Long-term: Intervention duration ≥8 weeks.

g LLM: large language model.

h Not applicable (fewer than 3 studies included).

i Rule: rule-based chatbot.

**Table 4.** Comparison of intervention time between groups.

Outcome and type^a^		Short-term^b^,^c^ (β; *P*)	Medium-term^d^ (β; *P*)	Long-term^e^ (β; *P*)
Depressive	
LLM^f^		0.245; —^g^	0.256; —	0.125; —
Rule^h^		0.036; .73	0.34; .04	0.373; .16
Anxiety	
LLM		0.817; .24	−0.265; —	−0.298; —
Rule		−0.11; .83	0.359; .05	0.181; .41

a Type: chatbot intervention type.

b Reference group: short-term.

c Short-term: intervention duration＜ 4 weeks.

d Medium-term: intervention duration 4-8 weeks.

e Long-term: intervention duration ≥8 weeks.

f LLM: large language model.

g Not applicable (fewer than 3 studies included).

h Rule: rule-based chatbot.

#### Participant Age

Subgroup analysis (see Table 5), intergroup comparison (see Table 6), and meta-regression analysis were used to analyze participant age.

In terms of improvement in depressive symptoms, the results of LLM-based interventions were not reliable due to the limited number of studies. In contrast, rule-based interventions showed a minor but not significant trend for both young people (*g*=0.294) and middle-aged people (*g*=0.230). Intergroup comparisons also did not reveal significant intervention effects for any particular demographic.

The different interventions had no significant effect on anxiety symptoms across the 3 age groups. The rule-based had a small, but nonsignificant, positive effect on anxiety symptoms in young people (*g*=0.244) and middle-aged people (*g*=0.165). No significant differences were found between the groups across time periods.

The meta-regression results also indicated that participant age did not significantly influence the effect size. Subgroup analyses with fewer than three studies were not performed. This suggests that individuals across different age groups show limited variation in their responses to LLM-based or rule-based interventions. The intervention effects are more likely influenced by other moderating variables rather than age itself.

**Table 5.** Subgroup analysis of age.

Outcome and type^a^		Young^b^ (*g*^c^; *P*; k^d^; 95% CI)	Medium^e^ (*g*; *P*; k; 95% CI)	Old^f^ (*g*; *P*; k; 95% CI)
Depressive	
LLM^g^		0.245; —^h^; 1; —	0.518; —; 2 —	−0.036; —; 1; —
Rule^i^		0.294; .32; 4; (−0.701 to 1.29)	0.23; .12; 3; (−0.173 to 0.632)	0.236; —; 1; —
Anxiety	
LLM		0.216; —; 2; —	1.16; —; 2; —	0.314; —; 1; —
Rule		0.244; .19; 4; (−0.27 to 0.759)	0.165; .35; 3; (−0.488 to 0.818)	−0.159; —; 1; —

a Type: chatbot intervention type.

b Young: participants < 30 years old.

c *g*: Hedges *g*.

d k: number of studies.

e Middle: participants aged 30-50 years.

f Old: participants aged >50 years.

g LLM: large language model.

h Not applicable (fewer than 3 studies included).

i Rule: rule-based chatbot.

**Table 6.** Comparison between intervention age groups.

Outcome and type^a^		Young^b^,^c^ (β; *P*)	Medium^d^ (β; *P*)	Old^e^ (β; *P*)
Depressive	
LLM^f^		0.245; —^g^	0.273; —	−0.281; —
Rule^h^		0.284; .34	−0.035; .89	−0.048; —
Anxiety	
LLM		0.245; —	0.890; —	−0.111; —
Rule		0.244;	−0.069; .72	−0.39; —

a Type: chatbot intervention type.

b Reference group: young people.

c Young: participants <30 years old.

d Middle: participants aged 30-50 years.

e Old: participants aged >50 years.

f LLM: large language model.

g Not applicable (fewer than 3 studies included).

h Rule: rule-based chatbot.

### Component Analysis

There are fundamental differences between rule-based and LLM-based chatbots in terms of underlying architecture, interaction patterns, and capability boundaries, which directly shape their distinct user experiences and application prospects. However, traditional meta-analyses often evaluate these heterogeneous interventions as a holistic “technology package,” with the core limitation being the difficulty in revealing which specific “active components” drive efficacy and how these components optimally align with different user characteristics and clinical scenarios.

To overcome this limitation and gain deeper insights into the mechanisms at work, this study introduces a component-level analysis in addition to conventional meta-analytic comparisons. We deconstruct chatbot systems into 4 core components: technology, therapy, implementation, and engagement, aiming to systematically compare the functional composition of the 2 types of chatbots. The goal is to identify key technological features or user elements significantly associated with efficacy, thereby providing empirical evidence for future precision design and optimization.

#### Technical Components

Technical components refer to a series of key technological blocks and implementation schemes that form the core conversational abilities of chatbots, determining their functional characteristics and application boundaries. This article mainly compares 2 dimensions: foundational capabilities and domain adaptation. Foundational capabilities concern the underlying architecture and knowledge sources of the system, while domain adaptation focuses on optimization and constraint mechanisms for specific scenarios.

Rule-based chatbots (Fido, X2 AI and Tess, SWPS University) rely on predefined workflows and natural language understanding technologies, which allow them to ensure safety and control by restricting generative capabilities. They also integrate structured intervention content, like cognitive behavioral therapy (CBT). These fixed intervention workflows and content are often manually authored and updated by psychology experts. In contrast, LLM-based chatbots are built on either proprietary or open-source large models, supporting open-domain free dialogue. These systems are typically trained on massive amounts of data and optimized using therapeutic conversation corpora, reinforcement learning with human feedback, or system prompt engineering to ensure their outputs meet ethical and contextual requirements [52]. Therefore, from a technological implementation perspective, if a team faces dual constraints of psychological expertise and model fine-tuning capability, using mature large model APIs would be a more feasible and economical option.

#### Treatment Components

Therapeutic components represent the digital implementation of psychological techniques, referring to the transformation of professional psychological strategies into interactive, executable conversational experiences. These components are direct factors influencing the effectiveness of interventions and can be specifically divided into four parts: evidence-based therapeutic integration, therapeutic alliance, crisis response, and personalized algorithms.

Rule-based chatbot therapeutic components are highly structured. Their evidence-based therapeutic integration is realized through standardized dialogue processes that guide users to complete intervention steps to the greatest extent possible. However, the templated and rigid nature of the responses can hinder the establishment of a therapeutic alliance [53]. Additionally, these chatbots lack the ability to provide personalized responses and rely on predefined keywords to trigger crisis intervention processes. In contrast, LLM-based chatbots have learned from extensive training data across a variety of psychotherapeutic approaches, including CBT, dialectical behavior therapy (DBT), and psychoanalysis. They can adjust intervention processes and depth based on user expressions, thereby building a more robust therapeutic alliance through multiturn dialogues. Their core advantage lies in personalized algorithms that can intelligently identify crisis risks and continuously optimize response strategies based on dialogue history, making users more likely to perceive warmth and empathy. However, precisely activating their therapeutic effectiveness remains a challenge.

Based on the characteristics of therapeutic components, a rule-based chatbot is best when an intervention needs to be standardized and structured. This applies to situations like verifying a program’s effectiveness or dealing with a specific psychological issue. On the other hand, if intervention goals are complex and dynamic, and there are no high expectations for treatment outcomes, an LLM chatbot is the better choice.

#### Implementation Components

Implementation components refer to the key operational factors that influence the transformation of technical solutions into practical services. This article compares the implementation approaches of 2 types of chatbots in terms of human-machine collaboration mode, system integration method, operational cost, and applicable scenarios.

Rule-based chatbots have all dialogue paths precoded by experts. Although their development and maintenance costs are relatively high, their outputs are highly deterministic, allowing for seamless and stable integration with traditional health care systems. This makes them particularly suitable for scenarios that require professional rigor and short-term intervention [54]. In contrast, LLM-based chatbots can quickly acquire empathetic capabilities by leveraging model APIs; however, their outputs are uncertain, necessitating additional adaptation layers for safe integration into health care systems. In terms of operational costs, LLM-based chatbots have a significant advantage. For example, using GPT-4 (OpenAI), a ten-round conversation of approximately 1000 words costs only about US$0.03. Meanwhile, fine-tuning a mature large model typically costs between tens and thousands of dollars, though these costs are decreasing with technological advancements [55]. Thus, they are particularly suited for scenarios where psychological resources are limited and rapid deployment is required.

Therefore, rule-based chatbots trade higher upfront development and ongoing maintenance costs for high certainty, security, and system compatibility in specialized scenarios, making them ideal for short-term, rigorous interventions. LLM-based chatbots, on the other hand, offer extremely low marginal costs and rapid deployment, making them more suitable for rapid rollout in resource-constrained, fault-tolerant, and inclusive support scenarios.

#### Engagement Components

Engagement components refer to a series of design elements that directly influence user willingness to use, depth of interaction, and long-term adherence, with the core goal of enhancing the attractiveness and sustainability of the service. This paper evaluates the engagement components of chatbots across 5 dimensions: interface design, user experience, interaction frequency, incentive features, and adherence monitoring.

Rule-based chatbots guide conversations through structured menus, ensuring interactive safety but also being prone to templated responses. They typically rely on scheduled email reminders and gamified external incentives to ensure frequent and regular user interactions and can quantitatively record conversation data, such as button clicks and conversation frequency. In contrast, LLM chatbots establish emotional connections through open, personified dialogue. Research results show a lower dropout rate than average (12%<15%), indicating that interactions are largely driven by user needs and rely on internal motivation to maintain engagement. Furthermore, data recording tools are needed to ensure the storage of conversation data.

Thus, from a user engagement perspective, rule-based chatbots are suitable for scenarios with standardized processes and strong guidance, such as those requiring cognitive adjustment and habit formation. LLM chatbots, on the other hand, are more suitable for users who are proactive in engaging and can provide emotional relief services.

## Discussion

### Principal Findings

This study examines related articles published between 2020 and 2025, systematically evaluating the effectiveness of LLM-based chatbots in the intervention of depressive and anxiety symptoms. It also compares their effectiveness with traditional rule-based chatbots, providing comprehensive evidence for the application of AI in the mental health field. Overall, the findings indicate that chatbots achieve a small effect size in improving depressive symptoms (*g*=0.283; *P*=.01). The effect size for improving anxiety symptoms is slightly higher but does not reach statistical significance (*g*=0.333; *P*=.07). Further analysis found that rule-based chatbots had a small but significant improvement in depressive symptoms and were more effective in settings with limited mental resources. For depression and anxiety symptoms, interventions lasting 4‐8 weeks may be more effective. However, due to the limited number of studies available, no reliable conclusions have been drawn regarding LLM-based chatbots.

### Chatbot

Current chatbot technologies are primarily divided into two categories: rule-based systems and those based on LLMs. These 2 types exhibit significant differences in performance and user experience. Traditional rule-based chatbots rely on structured intervention frameworks, keyword matching, and predefined corpora to generate responses. While this ensures control over the content of replies, it also brings notable limitations: the interaction patterns are highly fixed and predictable, making it difficult to adapt to the personalized needs of users. When faced with nonstandard, ambiguous, or complex user inputs, these systems often struggle with intent recognition, leading to failures in understanding or ineffective responses [56]. The system’s performance is directly constrained by the quality and size of the corpus—if the corpus is not rich, specialized, or outdated, the quality, relevance, and diversity of responses are significantly compromised. These inherent shortcomings directly translate into negative user experiences in practice, such as communication barriers due to failed intent recognition, insufficient accuracy of responses, unnatural and stiff expressions, high repetition of information, and an inability to provide personalized care. Ultimately, this often results in generic, insubstantial comforting phrases [57].

In contrast, generative chatbots based on LLMs have largely overcome the aforementioned issues by pretraining on vast and diverse text datasets, which endows them with a deep understanding and generation capability in natural language. These systems exhibit greater flexibility and contextual adaptability, enabling them to effectively parse and handle more complex and variable user inputs. Their robust long-context memory capability supports more coherent and in-depth multiturn dialogues [58]. More importantly, they are capable of generating highly contextualized and personalized responses based on conversation history and user characteristics, significantly enhancing user engagement and satisfaction. Positive user feedback often highlights the empathy level, comprehensiveness, and clarity of expression in responses. However, this open-domain dialogue system also presents new challenges, as it cannot guarantee that model outputs align with professional counseling ethics, posing various potential risks [59]. Therefore, carefully designed prompt engineering or targeted fine-tuning techniques to effectively constrain model behavior are essential. These measures ensure the safety, harmlessness, and ethical compliance of the outputs, making them critical elements and pressing issues that need addressing.

### Intervention Outcomes

Existing studies on the effectiveness of AI chatbots for mental health interventions show some discrepancies, reflecting the diversity and complexity of research methodologies in this field. A large-sample analysis by Li et al [60] indicates that AI interventions have limited effects on improving depression and anxiety symptoms, whereas studies by Villarreal-Zegarra et al [61] report significant small to moderate effects. Similarly, a meta-analysis that included 29 RCTs also found small to moderate improvements in depression and anxiety symptoms [62]. These differences may be attributed to several key factors: first, varying operational definitions of “chatbot” across studies, which include highly structured CBT dialogue systems and open-ended emotional support systems, leading to variability in intervention effects. Second, differences in research methodologies, such as the choice of assessment tools, timing of measurements, and types of control groups, can also influence results. Finally, differences in user group characteristics may underlie the inconsistency in research outcomes. The same intervention may have opposite effects in different cultural contexts; for example, the use of Tess significantly reduced depression and anxiety symptoms among American college students after 2 weeks, but showed no improvement among Argentinian college students [39,63].

It is noteworthy that direct comparisons of effect sizes between rule-based and LLM-based chatbots for depression or anxiety did not reach statistical significance. However, the imbalance in the number of studies included in the meta-analysis (6 LLM-based vs 9 rule-based) somewhat weakens the strength of direct comparison conclusions. This underscores that it is premature to assert that one technology is “universally superior” to the other; improvements may heavily depend on the design of the intervention program and the individual characteristics of the participants. The uncertainty regarding the effectiveness of LLM-based chatbots is mainly limited by the small sample size in the meta-analysis [64]. Larger, more rigorously designed, more balanced, and consistent studies are needed to validate their impact on mental health improvement.

Future research could explore in greater detail the interactive effects of different technological features (such as the degree of structure, conversational flexibility, and modes of empathetic expression) with specific symptom dimensions (such as cognitive sluggishness in depression and heightened vigilance in anxiety). Additionally, to address the challenges LLMs face in providing deep, consistent, and safe evidence-based interventions—such as ethical guidelines and the risk of hallucinations [65]—future studies could explore methods, such as prompt engineering, to effectively and ethically integrate LLM capabilities into evidence-based psychological intervention frameworks.

### Subgroup Analysis Conclusions

Through a multidimensional subgroup analysis, the study reveals the differential performance of chatbots across various control group types, intervention durations, and age groups. This provides crucial insights into understanding the boundaries of their effectiveness and optimizing application strategies.

In the context of depression interventions, rule-based chatbots significantly outperformed the blank control group but did not surpass bibliotherapy. This highlights that their effectiveness primarily stems from the “active intervention” facilitated by a structured interactive framework rather than merely conveying “therapeutic information.” This aligns well with the core principles of CBT, which involve helping users identify and modify detrimental thought and behavior patterns through structured interaction, feedback, and practice [66]. This advantage was not observed in anxiety interventions, which may be related to the different pathological mechanisms and intervention response characteristics of anxiety and depression [67]. Depression is often accompanied by low motivation and cognitive sluggishness, necessitating structured behavioral activation and cognitive guidance from external sources. In contrast, anxiety symptoms are more variable and have higher arousal, possibly requiring greater adaptability and flexibility in intervention formats. Rule-based systems appear limited when dealing with complex and acute anxiety-triggering situations [68]. Simultaneously, the limited number of studies on LLM chatbots presents clear limitations in the stability of results, preventing definitive conclusions regarding their intervention effectiveness. Future research should verify the intervention effects of LLM systems with larger sample sizes. Additionally, it is important to deconstruct the core active components driving efficacy in rule-based systems, such as specific interaction frequencies or personalized feedback mechanisms, and explore how these components can be effectively integrated into LLM systems to enhance their intervention depth and sustainability.

The analysis of intervention duration suggests a more complex picture than the traditional assumption that “longer is always better.” The data indicate a potential critical window of effectiveness for rule-based chatbots in treating depression and anxiety, with the greatest improvements observed within the 4- to 8-week period. Specifically, rule-based systems for depression demonstrated gradual benefits over time, which aligns with the principle that psychological skills require continuous practice to be effectively internalized [69]. For instance, improving homework adherence could further enhance the clinical efficacy of CBT. In contrast, due to the limited number of studies on LLM-based chatbots, it is difficult to draw definitive conclusions about the relationship between intervention effectiveness and duration. However, meta-regression results indicate that their intervention effectiveness does not show a significant trend over time. Some studies suggest that the lack of a systematic intervention framework may lead LLM chatbots to face adherence challenges in later stages of intervention, which could also hinder their long-term effectiveness [70].

Age subgroup analyses revealed no significant differences between the two interventions in improving symptoms of depression and anxiety, indicating a similar effect of chatbots across different age groups. This aligns with some previous research findings. Chocarro et al [71], found that the willingness to use AI technology does not increase with age, Grassini and Ree [72] and Kaya et al [73], also confirmed that age does not predict attitudes toward AI. However, some studies suggest that older adults have a higher acceptance of AI compared to younger individuals, although the majority of literature suggests that younger people tend to have more positive attitudes toward AI [74]. These conflicting findings highlight the need for more age-specific statistical data to facilitate interpretation. These insights further suggest that to maximize usability and effectiveness, intervention design must be tailored to the unique needs of different demographic groups. For instance, simplifying operational procedures could enhance engagement for older adults, while offering options for deeper exploration and genuine connection may be more appealing to younger users.

### Optimization Direction

A component analysis reveals that the 2 chatbot types are not simply iterations of technology but rather heterogeneous solutions with distinct strengths in technological foundations, therapeutic logic, implementation paths, and engagement models. In the complex field of mental health, there is no universally “optimal” technology; rather, there are “adaptive” designs that match specific clinical goals, user groups, and resource constraints. Therefore, the key bottleneck in the field has shifted from technical validation to systematically configuring these components to address multiple, even conflicting, real-world objectives.

To better guide future development, selection, and resource allocation, this article incorporates an optimization evaluation based on the EASE (European Association of Science Editors) framework, providing a comprehensive and systematic evaluation of chatbots across 4 dimensions: effectiveness, affordability, scalability, and efficiency. Furthermore, starting from the three phases of preparation, optimization, and evaluation, this article explores the strengths and weaknesses of each component, providing a basis for decision-making on precise and sustainable digital mental health interventions.

#### Preparation Phase

The preparation phase is the cornerstone of digital mental health interventions. It aims to systematically plan the chatbot based on clinical goals, user needs, and practical constraints.

Rule-based chatbots rely on logic trees and state machines, making deployment and operation costs manageable. However, their development relies heavily on professional clinical psychologists, and their exhaustive dialogue logic leads to poor maintenance and scalability, which in turn increases subsequent development and maintenance costs. LLM-based chatbots, on the other hand, generally enable interaction through model API calls, eliminating the need for deep engagement from psychologists. Later iterations can be achieved through methods like optimizing prompts, keeping overall development and maintenance costs manageable.

Therefore, if clinical safety and fidelity are primary considerations, the program should focus on building a systematic, standardized, and mature onboarding process to ensure consistent and reliable intervention for all users. If faced with cost and resource constraints, the intervention population is highly heterogeneous, and content needs to be continuously expanded, the program should prioritize long-term user experience and retention. Through empathy and personalized interactions, a strong therapeutic alliance can be established to achieve sustainable intervention results.

#### Optimization Phase

The optimization phase is the core of digital mental health interventions. Its goal is to achieve the optimal balance within the EASE framework by carefully configuring the chatbot’s internal components and collaborative methods.

First, the therapeutic alliance, a key predictor of the effectiveness of psychological interventions, relies on elements such as empathy, congruence, and positive regard. LLM can dynamically adjust conversational strategies, tone, and content depth based on the user’s real-time emotional state, cognitive level, and language expression habits, thereby fostering a deeper therapeutic alliance. Research by Nye et al [75] also demonstrates that personalized interventions have greater therapeutic effectiveness than standardized interventions, particularly in adolescents and those with depression.

Second, research indicates that users’ trust in and willingness to use chatbots largely depends on their perception of the bot’s capabilities and warmth [76]. Templated, mechanical responses in standardized interventions can easily undermine user engagement and compliance. Therefore, during the optimization phase, efforts should be focused on enhancing the naturalness of conversations and the accuracy of emotional responses. Through more humane and targeted interactions, users’ sense of trust and connection can be strengthened, thereby enhancing the sustained effectiveness of the intervention. Finally, while human oversight is the cornerstone of ethical safety, such as pre-set risk word triggers to prevent crises, it also limits affordability and user experience. Therefore, leveraging LLM capabilities and introducing a dynamic risk assessment model is a viable solution. This machine-generated early warning process significantly improves system scalability while ensuring safety.

#### Evaluation Phase

Previous studies have found that satisfaction and engagement with AI-powered counseling are significantly lower than those with real-person counseling. Even when provided with identical responses, users still prefer real-person counselors, highlighting the continuing gap between the two [77]. When responding to questions, AI is more likely to use supportive intervention strategies, with the core goal of fostering user self-insight, while real-person counselors tend to provide direct advice and factual information [78]. When AI engages in appropriate self-disclosure, it can enhance user intimacy and enjoyment [79]. Therefore, efforts to imbue chatbots with a sense of “personality” or “experience,” shifting the conversation from “response” to “companionship,” can foster long-term, stable emotional connections.

Meta-analysis results suggest that hybrid chatbot architectures offer a solution, to some extent. When users require cognitive adjustment, institutional guidance, or the processing of precise factual information, providing standardized intervention plans can ensure accurate responses and treatment fidelity. LLM, on the other hand, focuses on understanding user input and incorporating it into textual knowledge bases, generating more precise, personalized, and human-like responses, meeting the needs of a wider range of domains while maintaining cost-effectiveness. Existing research has shown that this hybrid chatbot model outperforms independent models, with an accuracy rate of 97.57% and significantly improved user satisfaction [80].

### Research Significance

This study systematically reviews research conducted over the past years on AI-based psychological interventions, examining the effectiveness of AI chatbots in improving symptoms of depression and anxiety. It also compares the effectiveness of traditional rule-based systems with LLM-based systems, as well as their moderating variables. This study holds significant innovative and practical value.

First, this study systematically reviews the development of AI chatbots in the field of mental health, which has positive implications for improving service accessibility and quality. In recent years, mental health issues have become increasingly prevalent, but there is a severe shortage of professional psychological service providers, making it difficult to meet the demand for high-quality care [81]. AI-based digital mental health services, with their convenience, low cost, and timely response, have become a key solution.

Second, by rigorously defining inclusion criteria, this study clearly distinguishes between the effectiveness of traditional rule-based and emerging LLM-based chatbots in psychological interventions. Despite the rapid development of generative AI, represented by ChatGPT (OpenAI), there is still a lack of systematic comparison of their actual effectiveness compared with mature rule-based systems. This study, leveraging a meta-analysis, is the first to empirically compare the intervention effectiveness of two technologies, revealing both the potential and limitations of LLM in terms of empathy, flexibility, and personalization. This distinction effectively reduces the heterogeneity caused by the mix of technologies in previous studies and provides a key basis for future technology selection and optimization.

Finally, the study systematically analyzes the two chatbot types based on the 4 components of technology, treatment, implementation, and engagement and proposes optimization strategies based on the three phases of preparation, implementation, and evaluation. Furthermore, subgroup analyses reveal differences in the effectiveness of rule-based and LLM chatbots across different control groups, intervention durations, and user age groups. This not only deepens our understanding of chatbot intervention mechanisms but also provides empirical evidence for building personalized and precise digital mental health service systems.

### Limitations and Future Directions

This study has certain limitations and shortcomings, (1) methodological limitations exist. Although mainstream databases were searched, systematic bulk retrieval of gray literature was not performed, which may lead to publication bias. The meta-analysis included only 15 studies, which is a relatively small number, limiting the statistical power and affecting the reliability of the conclusions. This is particularly true for studies related to LLMs, where the limited number of studies undermines the robustness of subgroup analysis results, requiring future validation through more high-quality research. (2) There is a high level of methodological heterogeneity among the included studies. Despite establishing uniform inclusion and exclusion criteria, significant differences remain in the design architecture, interaction frequency, and duration of different chatbots, necessitating a more cautious interpretation of the combined results. (3) The exploration of intervention mechanisms and core active components remains insufficient. Most studies primarily report overall intervention effects, lacking fine-grained analysis of which specific functions or interaction elements of the chatbots truly drive efficacy. (4) There is a lack of direct comparisons between AI interventions and standard psychological treatments, as well as systematic evaluations in real-world complex environments (eg, schools and hospitals), limiting the generalizability of the results to practical scenarios. (5) Finally, the role and effect of “therapeutic homework,” a core element in traditional evidence-based psychotherapy, cannot be examined in the context of AI interventions. How it is assigned, guided, provided with feedback, and assessed in human-computer interactions remains an important yet underexplored area.

Future research can expand and deepen in multiple directions, (1) diversified research scope: the scope of research can be appropriately expanded to include positive psychological indicators, such as well-being and psychological resilience. Enhancing the quantity and quality of the included literature will further validate the broad applicability of related AI technologies such as LLM. Additionally, exploring intervention mechanisms in complex mental health issues would be beneficial. (2) High-quality research literature: more methodologically rigorous and high-quality studies need to be included, particularly those using strict randomized controlled designs with sufficient statistical power, to address the current uncertainty in research conclusions caused by insufficient sample sizes. (3) Core component comparison: in-depth analysis and comparison of the core components of different AI systems will help to understand which elements are most effective in improving mental health. This knowledge can assist developers in better optimizing chatbot designs by integrating the most effective components, thereby enhancing their overall intervention capabilities. (4) Methodological transparency and reporting standards: present studies often lack systematic reporting on aspects such as the composition of training data, update timelines, and fine-tuning methods for domain-specific models, with technical details like prompt strategies also frequently omitted. Future research should promote more comprehensive reporting standards and enhance transparency regarding model development processes, data sources, and prompt engineering strategies to improve the reproducibility and comparability of findings. (5) Focus on methodological innovation: in light of the developmental needs in the digital health domain, it is essential to develop optimization methods that accommodate the rapidly evolving characteristics of AI technology. This involves clearly defining the division of roles and responsibilities between AI systems and human therapists in the intervention process. Moreover, empirical research should be conducted to validate the efficacy of different collaboration models, thereby enhancing the adaptability, scalability, and practical utility of AI-based psychological interventions. This approach will provide scientific evidence to refine human-machine collaborative mental health service models.

### Conclusion

This systematic review and meta-analysis evaluated the effectiveness of two different types of artificial intelligence chatbots in alleviating depressive and anxiety symptoms. The results indicated that rule-based chatbots exert a modest effect on improving depressive symptoms and are particularly superior to the blank control group, making them suitable for application in environments with limited psychological resources (eg, remote areas and primary care settings). Additionally, an intervention duration of 4–8 weeks may represent a critical window for optimizing the therapeutic effect of rule-based chatbots. Subgroup analyses further revealed that intervention duration and participant age did not significantly moderate the effectiveness of chatbot interventions. However, regarding LLM-based chatbots, robust evidence supporting their effectiveness remains lacking due to the small sample size of included studies. Therefore, future research should prioritize expanding the sample size of LLM-based chatbot interventions, conducting high-quality randomized controlled trials, and exploring the moderating factors (e.g., intervention intensity, participant characteristics) that may influence intervention outcomes. This study provides preliminary insights for the clinical application of two different types of AI chatbots in mental health care and highlights the need for more rigorous research to validate their effectiveness.

## Supplementary material

10.2196/78186 Multimedia Appendix 1 Search Strategy.

10.2196/78186 Multimedia Appendix 2 Data Coding Table.

10.2196/78186 Multimedia Appendix 3 Subgroup analyses Figures.

10.2196/78186 Checklist 1 PRISMA 2020 checklist.

## Abbreviations

AI: artificial intelligence
CBT: cognitive behavioral therapy
CNKI: China National Knowledge Infrastructure
Cochrane RoB 2: Cochrane Risk of Bias 2
CONSORT-AI: Consolidated Standards of Reporting Trials – Artificial Intelligence extension
EASE: European Association of Science Editors
HADS: Hospital Anxiety and Depression Scale
LLM: large language model
NLP: natural language processing
PHQ-9: Patient Health Questionnaire-9
PICOTS: population, intervention, comparator, outcome, timing, and study design
PRISMA: Preferred Reporting Items for Systematic Reviews and Meta-Analyses
PROSPERO: International Prospective Register of Systematic Reviews
RCT: randomized controlled trial
REML: Restricted Maximum Likelihood
ROBUST: Randomized, Open-Label, Blinded Endpoint Study
RVE: robust variance estimation
SMD: standardized mean difference
STAI: State-Trait Anxiety Inventory

## References

[1] (2022). World Mental Health Report: transforming mental health for all. World Health Organization.

[2] Moreno-Agostino D, Wu YT, Daskalopoulou C, Hasan MT, Huisman M, Prina M (2021). Global trends in the prevalence and incidence of depression:a systematic review and meta-analysis. J Affect Disord.

[3] Butryn T, Bryant L, Marchionni C, Sholevar F (2017). The shortage of psychiatrists and other mental health providers: causes, current state, and potential solutions. Int J Acad Med.

[4] Lehtimaki S, Martic J, Wahl B, Foster KT, Schwalbe N (2021). Evidence on digital mental health interventions for adolescents and young people: systematic overview. JMIR Ment Health.

[5] Andersson G (2016). Internet-delivered psychological treatments. Annu Rev Clin Psychol.

[6] Vrontis D, Christofi M, Pereira V, Tarba S, Makrides A, Trichina E (2022). Artificial intelligence, robotics, advanced technologies and human resource management: a systematic review. Int J Hum Resour Manag.

[7] Ke L, Tong S, Cheng P, Peng K (2025). Exploring the frontiers of LLMs in psychological applications: a comprehensive review. Artif Intell Rev.

[8] Lin CC, Huang AYQ, Yang SJH (2023). A review of AI-driven conversational chatbots implementation methodologies and challenges (1999–2022). Sustainability.

[9] Yang Q, Cheung K, Zhang Y, Zhang Y, Qin J, Xie YJ (2025). Conversational agents in physical and psychological symptom management: a systematic review of randomized controlled trials. Int J Nurs Stud.

[10] Kim JK, Chua M, Rickard M, Lorenzo A (2023). ChatGPT and large language model (LLM) chatbots: the current state of acceptability and a proposal for guidelines on utilization in academic medicine. J Pediatr Urol.

[11] Mahood Q, Van Eerd D, Irvin E (2014). Searching for grey literature for systematic reviews: challenges and benefits. Res Synth Methods.

[12] Aknin LB, De Neve JE, Dunn EW (2022). Mental health during the first year of the COVID-19 pandemic: a review and recommendations for moving forward. Perspect Psychol Sci.

[13] Appleton R, Williams J, Vera San Juan N (2021). Implementation, adoption, and perceptions of telemental health during the COVID-19 pandemic: systematic review. J Med Internet Res.

[14] Spytska L (2025). The use of artificial intelligence in psychotherapy: development of intelligent therapeutic systems. BMC Psychol.

[15] Olawade DB, Wada OZ, Odetayo A, David-Olawade AC, Asaolu F, Eberhardt J (2024). Enhancing mental health with artificial intelligence: current trends and future prospects. J Med Surg Public Health.

[16] Moher D, Liberati A, Tetzlaff J, Altman DG, PRISMA Group (2009). Preferred reporting items for systematic reviews and meta-analyses: the PRISMA statement. PLoS Med.

[17] Santelli JS, Smith Rogers A, Rosenfeld WD (2003). Guidelines for adolescent health research. a position paper of the society for adolescent medicine. J Adolesc Health.

[18] Baltes PB, Lindenberger U, Staudinger UM (2007). Handbook of Child Psychology.

[19] Dam SK, Hong CS, Qiao Y, Zhang C (2024). A complete survey on LLM-based AI chatbots. ArXiv.

[20] Büchter RB, Weise A, Pieper D (2020). Development, testing and use of data extraction forms in systematic reviews: a review of methodological guidance. BMC Med Res Methodol.

[21] Song SY, Kim B, Kim I (2017). Assessing reporting quality of randomized controlled trial abstracts in psychiatry: adherence to CONSORT for abstracts: a systematic review. PLoS ONE.

[22] Quigley JM, Thompson JC, Halfpenny NJ, Scott DA (2019). Critical appraisal of nonrandomized studies-a review of recommended and commonly used tools. J Eval Clin Pract.

[23] Pustejovsky JE, Tipton E (2022). Meta-analysis with robust variance estimation: expanding the range of working models. Prev Sci.

[24] Gignac GE, Szodorai ET (2016). Effect size guidelines for individual differences researchers. Pers Individ Dif.

[25] Rodgers MA, Pustejovsky JE (2021). Evaluating meta-analytic methods to detect selective reporting in the presence of dependent effect sizes. Psychol Methods.

[26] Tipton E, Pustejovsky JE, Ahmadi H (2019). Current practices in meta-regression in psychology, education, and medicine. Res Synth Methods.

[27] Fernández-Castilla B, Aloe AM, Declercq L (2021). Estimating outcome-specific effects in meta-analyses of multiple outcomes: a simulation study. Behav Res Methods.

[28] Higgins JPT, Thompson SG, Deeks JJ, Altman DG (2003). Measuring inconsistency in meta-analyses. BMJ.

[29] Rothstein HR, Sutton AJ, Borenstein M (2005). Publication Bias in Meta‐Analysis: Prevention, Assessment and Adjustments.

[30] Nakagawa S, Lagisz M, Jennions MD (2022). Methods for testing publication bias in ecological and evolutionary meta‐analyses. Methods Ecol Evol.

[31] Jabir AI, Lin X, Martinengo L, Sharp G, Theng YL, Tudor Car L (2024). Attrition in conversational agent-delivered mental health interventions: systematic review and meta-analysis. J Med Internet Res.

[32] Yahagi M, Hiruta R, Miyauchi C, Tanaka S, Taguchi A, Yaguchi Y (2024). Comparison of conventional anesthesia nurse education and an artificial intelligence chatbot (ChatGPT) intervention on preoperative anxiety: a randomized controlled trial. J Perianesth Nurs.

[33] Wang Y, Li S (2024). Tech vs. tradition: ChatGPT and mindfulness in enhancing older adults’ emotional health. Behav Sci (Basel).

[34] Chen C, Lam KT, Yip KM (2025). Comparison of an AI chatbot with a nurse hotline in reducing anxiety and depression levels in the general population: pilot randomized controlled trial. JMIR Hum Factors.

[35] Kerimoglu Yildiz G, Turk Delibalta R, Coktay Z (2025). Artificial intelligence-assisted chatbot: impact on breastfeeding outcomes and maternal anxiety. BMC Pregnancy Childbirth.

[36] Zhang H, Wang X, Luo H (2025). Comparison of preoperative education by artificial intelligence versus traditional physicians in perioperative management of urolithiasis surgery: a prospective single-blind randomized controlled trial conducted in China. Front Med (Lausanne).

[37] Heinz MV, Mackin DM, Trudeau BM (2025). Randomized trial of a generative AI chatbot for mental health treatment. NEJM AI.

[38] Karkosz S, Szymański R, Sanna K, Michałowski J (2024). Effectiveness of a web-based and mobile therapy chatbot on anxiety and depressive symptoms in subclinical young adults: randomized controlled trial. JMIR Form Res.

[39] Klos MC, Escoredo M, Joerin A, Lemos VN, Rauws M, Bunge EL (2021). Artificial intelligence-based chatbot for anxiety and depression in university students: pilot randomized controlled trial. JMIR Form Res.

[40] Kannampallil T, Ajilore OA, Lv N (2023). Correction: Effects of a virtual voice-based coach delivering problem-solving treatment on emotional distress and brain function: a pilot RCT in depression and anxiety. Transl Psychiatry.

[41] MacNeill AL, Doucet S, Luke A (2024). Effectiveness of a mental health chatbot for people with chronic diseases: randomized controlled trial. JMIR Form Res.

[42] Liu H, Peng H, Song X, Xu C, Zhang M (2022). Using AI chatbots to provide self-help depression interventions for university students: a randomized trial of effectiveness. Internet Interv.

[43] He Y, Yang L, Zhu X (2022). Mental health chatbot for young adults with depressive symptoms during the COVID-19 pandemic: single-blind, three-arm randomized controlled trial. J Med Internet Res.

[44] Danieli M, Ciulli T, Mousavi SM (2022). Assessing the impact of conversational artificial intelligence in the treatment of stress and anxiety in aging adults: randomized controlled trial. JMIR Ment Health.

[45] Nicol G, Wang R, Graham S, Dodd S, Garbutt J (2022). Chatbot-delivered cognitive behavioral therapy in adolescents with depression and anxiety during the COVID-19 pandemic: feasibility and acceptability study. JMIR Form Res.

[46] Sabour S, Zhang W, Xiao X (2023). A chatbot for mental health support: exploring the impact of Emohaa on reducing mental distress in China. Front Digit Health.

[47] Hedges LV, Tipton E, Johnson MC (2010). Robust variance estimation in meta-regression with dependent effect size estimates. Res Synth Methods.

[48] Little RJA (1988). A test of missing completely at random for multivariate data with missing values. J Am Stat Assoc.

[49] Swift JK, Greenberg RP (2012). Premature discontinuation in adult psychotherapy: a meta-analysis. J Consult Clin Psychol.

[50] Torous J, Lipschitz J, Ng M, Firth J (2020). Dropout rates in clinical trials of smartphone apps for depressive symptoms: a systematic review and meta-analysis. J Affect Disord.

[51] Viechtbauer W (2005). Bias and efficiency of meta-analytic variance estimators in the random-effects model. J Educ Behav Stat.

[52] Smirnov E (2025). Enhancing qualitative research in psychology with large language models: a methodological exploration and examples of simulations. Qual Res Psychol.

[53] Wan Ab.Rahman WN, Abdul Hamid NM (2024). Rule-based chatbot for early self-depression indication: a promising approach. JOIV: Int J Inform Visualization.

[54] Thorat SA, Jadhav V (2020). A review on implementation issues of rule-based chatbot systems. SSRN Journal.

[55] Xia Y, Kim J, Chen Y Understanding the performance and estimating the cost of llm fine-tuning.

[56] Solomon E, Tilahun SL, Department of Software Engineering, Addis Ababa Science and Technology University, Ethiopia, Department of Mathematics, Physics, and Statistics; HPC and Big Data Analytics CoE, Addis Ababa Science and Technology University, Ethiopia (2024). Rule based chatbot design methods: a review. JCSDA.

[57] Raval H (2020). Limitations of existing chatbot with analytical survey to enhance the functionality using emerging technology. Int J Res Anal Rev.

[58] Yi Z, Ouyang J, Xu Z (2024). A survey on recent advances in LLM-based multi-turn dialogue systems. ACM Comput Surv.

[59] Wang J, Ma W, Sun P, Zhang M, Nie JY (2024). Understanding user experience in large language model interactions. arXiv.

[60] Li H, Zhang R, Lee YC, Kraut RE, Mohr DC (2023). Systematic review and meta-analysis of AI-based conversational agents for promoting mental health and well-being. NPJ Digit Med.

[61] Villarreal-Zegarra D, Reategui-Rivera CM, García-Serna J (2024). Self-administered interventions based on natural language processing models for reducing depressive and anxiety symptoms: systematic review and meta-analysis. JMIR Ment Health.

[62] Lau Y, Ang WHD, Ang WW, Pang PCI, Wong SH, Chan KS, Lamela D (2025). Artificial intelligence–based psychotherapeutic intervention on psychological outcomes: a meta‐analysis and meta‐regression. Depress Anxiety.

[63] Fulmer R, Joerin A, Gentile B, Lakerink L, Rauws M (2018). Using psychological artificial ntelligence (Tess) to relieve symptoms of depression and anxiety: randomized controlled trial. JMIR Ment Health.

[64] Kumar V, Srivastava P, Dwivedi A, Santosh KC, Makkar A, Conway M, Singh AK, Vacavant A, Abou El Kalam A, Bouguelia MR, Hegadi RS (2024). Recent Trends in Image Processing and Pattern Recognition.

[65] Banerjee S, Agarwal A, Singla S (2024). LLMs will always hallucinate, and we need to live with this. arXiv.

[66] Wright JH (2006). Cognitive behavior therapy: basic principles and recent advances. FOC.

[67] Babl A, Grosse Holtforth M, Perry JC (2019). Comparison and change of defense mechanisms over the course of psychotherapy in patients with depression or anxiety disorder: evidence from a randomized controlled trial. J Affect Disord.

[68] Lim SM, Shiau CWC, Cheng LJ, Lau Y (2022). Chatbot-delivered psychotherapy for adults with depressive and anxiety symptoms: a systematic review and meta-regression. Behav Ther.

[69] Quintana SM, Meara NM Internalization of therapeutic relationships in short-term psychotherapy. J Couns Psychol.

[70] Scholich T, Barr M, Wiltsey Stirman S, Raj S (2025). A comparison of responses from human therapists and large language model-based chatbots to assess therapeutic communication: mixed methods study. JMIR Ment Health.

[71] Chocarro R, Cortiñas M, Marcos-Matás G (2023). Teachers’ attitudes towards chatbots in education: a technology acceptance model approach considering the effect of social language, bot proactiveness, and users’ characteristics. Educ Stud.

[72] Grassini S, Ree AS Hope or doom AI-ttitude? examining the impact of gender, age, and cultural differences on the envisioned future impact of artificial intelligence on humankind.

[73] Kaya F, Aydin F, Schepman A, Rodway P, Yetişensoy O, Demir Kaya M (2024). The roles of personality traits, AI anxiety, and demographic factors in attitudes toward artificial intelligence. Int J Hum Comput Interact.

[74] Tully CJ (2003). Growing up in technological worlds: how modern technologies shape the everyday lives of young people. Bull Sci Technol Soc.

[75] Nye A, Delgadillo J, Barkham M (2023). Efficacy of personalized psychological interventions: a systematic review and meta-analysis. J Consult Clin Psychol.

[76] Christoforakos L, Gallucci A, Surmava-Große T, Ullrich D, Diefenbach S (2021). Can robots earn our trust the same way humans do? A systematic exploration of competence, warmth, and anthropomorphism as determinants of trust development in HRI. Front Robot AI.

[77] Zhang WZ, Lian R (2025). Counselor type (Human/AI) and consultation intention: a moderated mediation model of trust and psychological counseling scenarios. BMC Psychol.

[78] Yonatan‐Leus R, Brukner H (2025). Comparing perceived empathy and intervention strategies of an AI chatbot and human psychotherapists in online mental health support. Couns and Psychother Res.

[79] Lee YC, Yamashita N, Huang Y, Fu W (2020). “I hear you, i feel you”: encouraging deep self-disclosure through a chatbot. https://dl.acm.org/doi/proceedings/10.1145/3313831.

[80] Naveen P, Haw SC, Nadthan D, Ramamoorthy SK (2023). Improving chatbot performance using hybrid deep learning approach. JSMS.

[81] Lu J, Xu X, Huang Y (2021). Prevalence of depressive disorders and treatment in China: a cross-sectional epidemiological study. Lancet Psychiatry.

[82] A systematic review and meta-analysis. GitHub.

