# Supplementary material for: The Efficacy of Rule-Based Versus Large Language Model-Based Chatbots in Alleviating Symptoms of Depression and Anxiety: Systematic Review and Meta-Analysis
Source: J Med Internet Res. 2025 Dec 4;27:e78186. doi: 10.2196/78186 (PMC12677872; doi:10.2196/78186)
Supplement: Multimedia Appendix 1 [file jmir-v27-e78186-s001.doc]

PubMed Search Strategy

| **ID** | **Hits** | **Search** |
| --- | --- | --- |
| #1 And  #2 And  #3 | 177 | (((((((((((((Psychological Well-Being[MeSH Terms]) OR (mental health[MeSH Terms])) OR (Personal Satisfaction[MeSH Terms])) OR (mental Hygiene[Title/Abstract])) OR (depressive disorder[Title/Abstract])) OR (depressive neurosis[Title/Abstract])) OR (mood disorders[Title/Abstract])) OR (anxiety[Title/Abstract])) OR (Angst[Title/Abstract])) OR (social anxiety[Title/Abstract])) OR (depression[Title/Abstract])) OR (dysthymia[Title/Abstract]) AND (y_5[Filter])) AND ((((((((((counseling[MeSH Terms]) OR (Distance Counseling[MeSH Terms])) OR (Cognitive Behavioral Therapy[MeSH Terms])) OR (mental Health Services[MeSH Terms])) OR (psychology[Title/Abstract])) OR (online counseling[Title/Abstract])) OR (chatbot counseling[Title/Abstract])) OR (online psychotherapy[Title/Abstract])) OR (digital psychotherapy[Title/Abstract])) OR (CBT[Title/Abstract]) AND (y_5[Filter]))) AND ((((((((((Generative Artificial Intelligence[MeSH Terms]) OR (Artificial intelligence[MeSH Terms])) OR (machine learning[MeSH Terms])) OR (Deep Learning[MeSH Terms])) OR (chatbot[Title/Abstract])) OR (Chat GPT[Title/Abstract])) OR (LLM[Title/Abstract])) OR (NLP[Title/Abstract])) OR (Machine Intelligence[Title/Abstract])) OR (Computer Reasoning[Title/Abstract]) AND ( Filters: from 2020/1/1 - 2025/7/21) |
| #3 | **749891** | (((((((((((Psychological Well-Being[MeSH Terms]) OR (mental health[MeSH Terms])) OR (Personal Satisfaction[MeSH Terms])) OR (mental Hygiene[Title/Abstract])) OR (depressive disorder[Title/Abstract])) OR (depressive neurosis[Title/Abstract])) OR (mood disorders[Title/Abstract])) OR (anxiety[Title/Abstract])) OR (Angst[Title/Abstract])) OR (social anxiety[Title/Abstract])) OR (depression[Title/Abstract])) OR (dysthymia[Title/Abstract]) |
| #2 | **234038** | (((((((((counseling[MeSH Terms]) OR (Distance Counseling[MeSH Terms])) OR (Cognitive Behavioral Therapy[MeSH Terms])) OR (mental Health Services[MeSH Terms])) OR (psychology[Title/Abstract])) OR (online counseling[Title/Abstract])) OR (chatbot counseling[Title/Abstract])) OR (online psychotherapy[Title/Abstract])) OR (digital psychotherapy[Title/Abstract])) OR (CBT[Title/Abstract]) |
| #1 | 250309 | (((((((((Generative Artificial Intelligence[MeSH Terms]) OR (Artificial intelligence[MeSH Terms])) OR (machine learning[MeSH Terms])) OR (Deep Learning[MeSH Terms])) OR (chatbot[Title/Abstract])) OR (Chat GPT[Title/Abstract])) OR (LLM[Title/Abstract])) OR (NLP[Title/Abstract])) OR (Machine Intelligence[Title/Abstract])) OR (Computer Reasoning[Title/Abstract]) |

Cochrane Library Search Strategy

| **ID** | **Hits** | **Search** |
| --- | --- | --- |
| #1 | 3606 | MeSH descriptor: [Artificial Intelligence] explode all trees |
| #2 | 1165 | MeSH descriptor: [Machine Learning] explode all trees |
| #3 | 380 | MeSH descriptor: [Deep Learning] explode all trees |
| #4 | 1076 | (Generative Artificial Intelligence) OR (chatbot) OR (Chat GPT) OR (LLM) OR (NLP) |
| #5 | 7542 | MeSH descriptor: [Counseling] explode all trees |
| #6 | 26 | MeSH descriptor: [Distance Counseling] explode all trees |
| #7 | 14691 | MeSH descriptor: [Cognitive Behavioral Therapy] explode all trees |
| #8 | 9521 | MeSH descriptor: [Mental Health Services] explode all trees |
| #9 | 126199 | (psychology) OR (online counseling) OR (chatbot counseling) OR (online psychotherapy) OR (digital psychotherapy) OR (CBT) |
| #10 | 75 | MeSH descriptor: [Psychological Well-Being] explode all trees |
| #11 | 3496 | MeSH descriptor: [Mental Health] explode all trees |
| #12 | 1648 | MeSH descriptor: [Personal Satisfaction] explode all trees |
| #13 | 164595 | (mental Hygiene) OR (depressive disorder) OR (depressive neurosis) OR (mood disorders) OR (anxiety) OR (Angst) OR (social anxiety) OR (depression) OR (dysthymia) |
| #14 | 4568 | #1 or #2 or #3 or #4 |
| #15 | 134002 | #5 or #6 or #7 or #8 or #9 |
| #16 | 167628 | #10 or #11 or #12 or #13 |
| #17 | 144 | #14 AND #15 AND #16 |
| 18 | 144 | 2020/1/1-2025/07/21 |

Scopus Search Strategy

| **Scopus** |
| --- |
| ( TITLE-ABS-KEY ( "Generative Artificial Intelligence" ) OR TITLE-ABS-KEY ( chatbot ) OR TITLE-ABS-KEY ( "Chat GPT" ) OR TITLE-ABS-KEY ( "Artificial intelligence" ) OR TITLE-ABS-KEY ( llm ) OR TITLE-ABS-KEY ( nlp ) OR TITLE-ABS-KEY ( "machine learning" ) OR TITLE-ABS-KEY ( "Machine Intelligence" ) OR TITLE-ABS-KEY ( "Computer Reasoning" ) OR TITLE-ABS-KEY ( "Deep Learning" ) ) AND ( TITLE-ABS-KEY ( counseling ) OR TITLE-ABS-KEY ( "Distance Counseling" ) OR TITLE-ABS-KEY ( psychology ) OR TITLE-ABS-KEY ( "online counseling" ) OR TITLE-ABS-KEY ( "chatbot counseling" ) OR TITLE-ABS-KEY ( "online psychotherapy" ) OR TITLE-ABS-KEY ( "digital psychotherapy" ) OR TITLE-ABS-KEY ( "Cognitive Behavioral Therapy" ) OR TITLE-ABS-KEY ( cbt ) ) AND ( TITLE-ABS-KEY ( "mental health" ) OR TITLE-ABS-KEY ( "Psychological Well-Being" ) OR TITLE-ABS-KEY ( "Personal Satisfaction" ) OR TITLE-ABS-KEY ( "Mental Hygiene" ) OR TITLE-ABS-KEY ( "Depressive Disorder" ) OR TITLE-ABS-KEY ( neurosis ) OR TITLE-ABS-KEY ( "Mood Disorders" ) OR TITLE-ABS-KEY ( anxiety ) OR TITLE-ABS-KEY ( angst ) OR TITLE-ABS-KEY ( "Social Anxiety" ) OR TITLE-ABS-KEY ( "Depressive Disorder" ) OR TITLE-ABS-KEY ( depression ) OR TITLE-ABS-KEY ( dysthymia ) ) AND PUBYEAR > 2019 AND PUBYEAR < 2026 |
| 2673 |

Embase Search Strategy

| **ID** | **Search** | **Hits** |
| --- | --- | --- |
| #31 | #30 AND [2020-2025]/py | 4,095 |
| #30 | #18 AND #29 | 4,709 |
| #29 | #19 OR #20 OR #21 OR #22 OR #23 OR #24 OR #25 OR #26 OR #27 OR #28 | 2,243,754 |
| #28 | 'dysthymia' | 10,913 |
| #27 | 'social anxiety' | 13,025 |
| #26 | 'anxiety' | 623,775 |
| #25 | 'mood disorder' | 67,344 |
| #24 | 'neurosis' | 55,664 |
| #23 | 'depression' | 1,013,507 |
| #22 | 'mental hygiene' | 8,844 |
| #21 | 'satisfaction' | 432,895 |
| #20 | 'mental health' | 733,246 |
| #19 | 'psychological well-being' | 53,428 |
| #18 | #10 AND #17 | 15,375 |
| #17 | #11 OR #12 OR #13 OR #14 OR #15 OR #16 | 1,643,353 |
| #16 | 'cognitive behavioral therapy' | 49,580 |
| #15 | digital AND psychotherapy | 2,966 |
| #14 | 'telepsychotherapy' | 664 |
| #13 | 'e-counseling' | 635 |
| #12 | 'psychology' | 1,351,669 |
| #11 | 'counseling' | 294,018 |
| #10 | #1 OR #2 OR #3 OR #4 OR #5 OR #6 OR #7 OR #8 OR #9 | 419,675 |
| #9 | 'automated reasoning' | 195 |
| #8 | 'deep learning' | 111,876 |
| #7 | 'machine learning' | 228,583 |
| #6 | nlp | 7,763 |
| #5 | llm | 3,806 |
| #4 | 'artificial intelligence' | 165,728 |
| #3 | 'chatgpt' | 8,621 |
| #2 | 'generative artificial intelligence'/exp OR 'generative artificial intelligence' | 11,719 |
| #1 | 'chatbot' | 3,872 |

PsycINFO Search Strategy

| **PsycINFO** |
| --- |
| 1,564 Results for (Any Field: "Generative Artificial Intelligence" OR Any Field: chatbot OR Any Field: "Chat GPT" OR Any Field: "Artificial intelligence" OR Any Field: LLM OR Any Field: NLP OR Any Field: "machine learning" OR Any Field: "Machine Intelligence" OR Any Field: "Computer Reasoning" OR Any Field: "Deep Learning") AND (Any Field: counseling OR Any Field: "Distance Counseling" OR Any Field: psychology OR Any Field: "online counseling" OR Any Field: "chatbot counseling" OR Any Field: "online psychotherapy" OR Any Field: "digital psychotherapy" OR Any Field: "Cognitive Behavioral Therapy" OR Any Field: CBT) AND (Any Field: "mental health" OR Any Field: "Psychological Well-Being" OR Any Field: "Personal Satisfaction" OR Any Field: "Mental Hygiene" OR Any Field: "Depressive Disorder" OR Any Field: Neurosis OR Any Field: "Mood Disorders" OR Any Field: anxiety OR Any Field: Angst OR Any Field: "Social Anxiety" OR Any Field: Depression OR Any Field: dysthymia)) AND Year: 2020/1/1-2025/07/21 |
| 1653 |

CNKI Search Strategy

| **中国知网** |
| --- |
| （篇关摘：AI + 人工智能 + 大语言模型 + 对话机器人(精确)）AND（篇关摘：AI心理咨询 + 数字疗法 + 在线心理咨询 + 聊天机器人 + 机器人(精确)）AND（篇关摘：焦虑 + 抑郁 + 心境恶劣 + 幸福感 + 心理健康(精确)）AND发表时间：2020/1/1-2025/07/21 |
| 132 |

WangFang Search Strategy

| **万方数据库** |
| --- |
| **(主题:((AI)or(人工智能) or(大语言模型)or(对话机器人)) and 主题:((AI心理咨询)or(数字疗法)or(在线心理咨询)or(聊天机器人)or(机器人)) and 主题:((焦虑)or(抑郁)or(心境恶劣)or(幸福感 )or(心理健康))) and 发表时间:2020-2025** |
| **183** |
