# Supplementary material for: The Efficacy of Rule-Based Versus Large Language Model-Based Chatbots in Alleviating Symptoms of Depression and Anxiety: Systematic Review and Meta-Analysis
Source: J Med Internet Res. 2025 Dec 4;27:e78186. doi: 10.2196/78186 (PMC12677872; doi:10.2196/78186)
Supplement: Multimedia Appendix 2 [file jmir-v27-e78186-s002.docx]

Data Coding Table

| **Publication Characteristics** | | **Intervention Characteristics** | | | | **Control Characteristics** | **Participant Characteristics** | | **Outcome Measures** | |
| --- | --- | --- | --- | --- | --- | --- | --- | --- | --- | --- |
| **Author(Year)** | **Country** | **Study Design** | **Type** | **Duration** | **g** |  | **Age** | **Attrition Rate** | **Psychological Traits** | **Scales** |
| Musashi Yahagi(2024) | Japan | RCT | LLM | Short | 0.134 | Human | Old | 15% | Anxiety | GAD-7 |
| Ying Wang(2024) | China | Quasi-experimental | LLM | Long | -0.036 | Book | Old | 50% | Depression | PHQ-9 |
| Chen Chen(2025) | China | RCT | LLM | Short | 0.245 | Human |  | 16.95% | Depression  Anxiety | PHQ-9  GAD-7 |
| Gizem Kerimoglu Yildiz(2025) | Türkiye | Quasi-experimental | LLM | Short | 0.339 | Book | Young | 16.70% | Anxiety | STAI |
| Hui Zhang(2025) | China | RCT | LLM | Short | 1.274 | Human | Middle | 0% | Anxiety | STAI |
| Michael V. Heinz, M.D(2025) | United States | RCT | LLM | Medium | 0.501 | Blank | Middle | 2.10% | Depression  Anxiety | PHQ-9  GAD-7 |
| Stanisław Karkosz(2024) | Poland | RCT | Rule | Short | 0.286 | Book | Young | 16.05% | Depression  Anxiety | PHQ-9  STAI |
| Maria Carolina Klos(2021) | Argentina | RCT | Rule | Long | 0.489 | Book |  | 72.38% | Anxiety | GAD-7 |
| Thomas Kannampallil(2023) | United States | RCT | Rule | Long | 0.484 | Blank | Middle | 4.76% | Depression  Anxiety | HANDS |
| A Luke MacNeill(2024) | United States | RCT | Rule | Short | 0.042 | Blank | Middle | 3.80% | Depression  Anxiety | PHQ-9  GAD-7 |
| Hao Liu(2022) | China | RCT | Rule | Long | 0.822 | Book | Young | 24.10% | Depression  Anxiety | PHQ-9  GAD-7 |
| Yuhao He(2022) | China | RCT | Rule | Short | 0.531 | Book | Young | 14.29% | Depression | PHQ-9 |
| Morena Danieli(2022) | Italy | RCT | Rule | Long | 0.53 | Human | Old | 6.67% | Depression   Anxiety | PHQ-9  GAD-7 |
| Ginger Nicol(2022) | United States | RCT | Rule | Medium | 0.924 | Blank | Young | 5.56% | Depression   Anxiety | PHQ-8 GAD-7 |
| Sahand Sabour(2023) | China | RCT | Rule | Short | 0.09 | Blank | Middle | 16.11% | Depression   Anxiety | PHQ-9  GAD-7 |

Type: Intervention type

LLM: Chatbot based on large language model

Rule: Rule-based chatbots

Control Characteristics: Human (Various psychological interventions provided by counselors, doctors or nurses); Book (Participants read psychology books or materials on their own); Blank (No intervention measures)

References for Studies Included in the Coding Table

1. Yahagi M, Hiruta R, Miyauchi C, Tanaka S, Taguchi A, Yaguchi Y. Comparison of Conventional Anesthesia Nurse Education and an Artificial Intelligence Chatbot (ChatGPT) Intervention on Preoperative Anxiety: A Randomized Controlled Trial. J Perianesth Nurs. Dec 2024; 39(5):767-771. PMID: 38520470
2. Wang Y, Li S. Tech vs. Tradition: ChatGPT and Mindfulness in Enhancing Older Adults' Emotional Health. Behav Sci (Basel). Oct 2024; 14(10):923. PMID: 39457795
3. Chen C, Lam KT, Yip KM, et al. Comparison of an AI Chatbot With a Nurse Hotline in Reducing Anxiety and Depression Levels in the General Population: Pilot Randomized Controlled Trial. JMIR Hum Factors. Mar 2025; 12:e65785. PMID: 40048637
4. Kerimoglu Yildiz G, Turk Delibalta R, Coktay Z. Artificial intelligence-assisted chatbot: impact on breastfeeding outcomes and maternal anxiety. BMC Pregnancy Childbirth. May 2025; 25(1):631. PMID: 40448061
5. Zhang H, Wang X, Luo H, et al. Comparison of preoperative education by artificial intelligence versus traditional physicians in perioperative management of urolithiasis surgery: a prospective single-blind randomized controlled trial conducted in China. Front Med (Lausanne). Jun 2025; 12:1543630. PMID: 40636362
6. Heinz M V, Mackin D M, Trudeau B M, et al. Randomized trial of a generative AI chatbot for mental health treatment. Nejm Ai. Mar 2025; 2(4): AIoa2400802. doi: 10.1056/AIoa2400802
7. Karkosz S, Szymański R, Sanna K, Michałowski J. Effectiveness of a Web-based and Mobile Therapy Chatbot on Anxiety and Depressive Symptoms in Subclinical Young Adults: Randomized Controlled Trial. JMIR Form Res. Mar 2024; 8:e47960. PMID: 38506892
8. Klos MC, Escoredo M, Joerin A, Lemos VN, Rauws M, Bunge EL. Artificial Intelligence-Based Chatbot for Anxiety and Depression in University Students: Pilot Randomized Controlled Trial. JMIR Form Res. 2021;5(8):e20678. Published 2021 Aug 12. doi:10.2196/20678
9. Kannampallil T, Ajilore OA, Lv N, et al. Correction: Effects of a virtual voice-based coach delivering problem-solving treatment on emotional distress and brain function: a pilot RCT in depression and anxiety. Transl Psychiatry. Jul 2023; 13(1):242. PMID: 37402748
10. MacNeill AL, Doucet S, Luke A. Effectiveness of a Mental Health Chatbot for People With Chronic Diseases: Randomized Controlled Trial. JMIR Form Res. May 2024; 8:e50025. PMID: 38814681
11. Liu H, Peng H, Song X, Xu C, Zhang M. Using AI chatbots to provide self-help depression interventions for university students: A randomized trial of effectiveness. Internet Interv. Jan 2022; 27:100495. PMID: 35059305
12. He Y, Yang L, Zhu X, et al. Mental Health Chatbot for Young Adults With Depressive Symptoms During the COVID-19 Pandemic: Single-Blind, Three-Arm Randomized Controlled Trial. J Med Internet Res. Nov 2022; 24(11):e40719. PMID: 36355633
13. Danieli M, Ciulli T, Mousavi SM, et al. Assessing the Impact of Conversational Artificial Intelligence in the Treatment of Stress and Anxiety in Aging Adults: Randomized Controlled Trial. JMIR Ment Health. Sep 2022; 9(9):e38067. PMID: 36149730
14. Nicol G, Wang R, Graham S, Dodd S, Garbutt J. Chatbot-Delivered Cognitive Behavioral Therapy in Adolescents With Depression and Anxiety During the COVID-19 Pandemic: Feasibility and Acceptability Study. JMIR Form Res. Nov 2022; 6(11):e40242. PMID: 36413390
15. Sabour S, Zhang W, Xiao X, et al. A chatbot for mental health support: exploring the impact of Emohaa on reducing mental distress in China. Frontiers in digital health. May 2023; 5: 1133987. PMID: 37214342
