# Supplementary material for: The Efficacy of Rule-Based Versus Large Language Model-Based Chatbots in Alleviating Symptoms of Depression and Anxiety: Systematic Review and Meta-Analysis
Source: J Med Internet Res. 2025 Dec 4;27:e78186. doi: 10.2196/78186 (PMC12677872; doi:10.2196/78186)
Supplement: Multimedia Appendix 3 [file jmir-v27-e78186-s003.docx]

## Multimedia Appendix 3


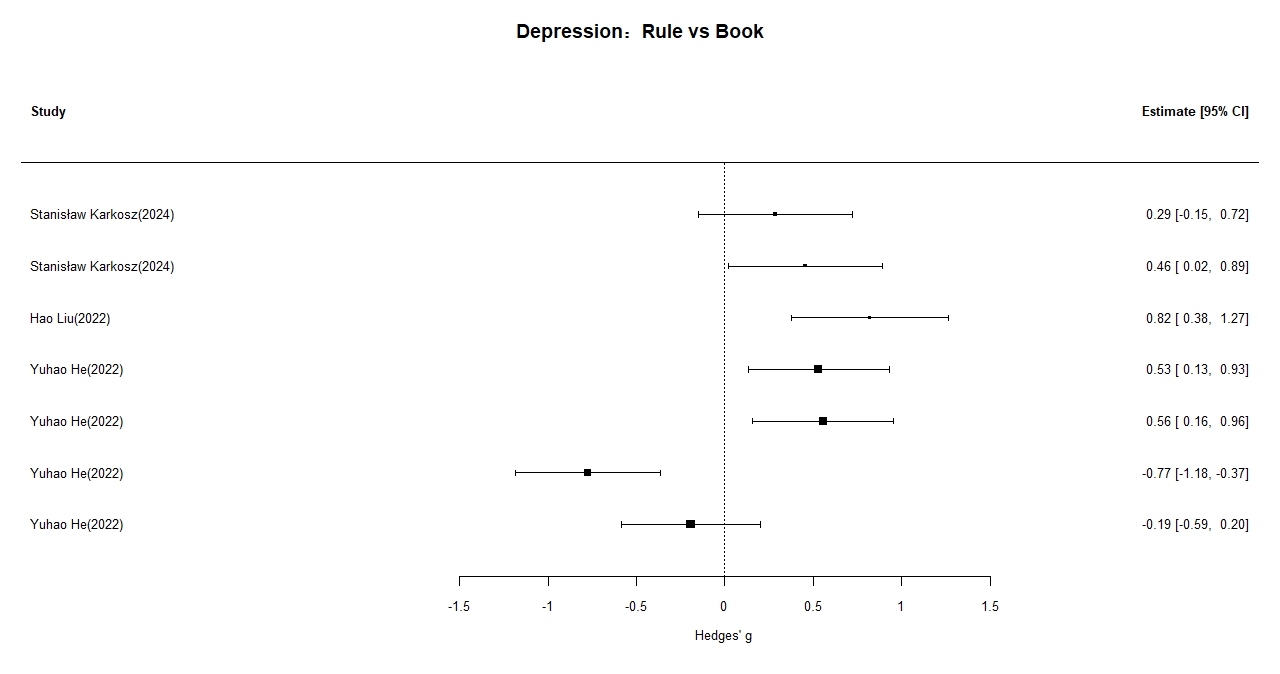


Figure S1. Forest Plot of Depression Interventions - Rule vs Book


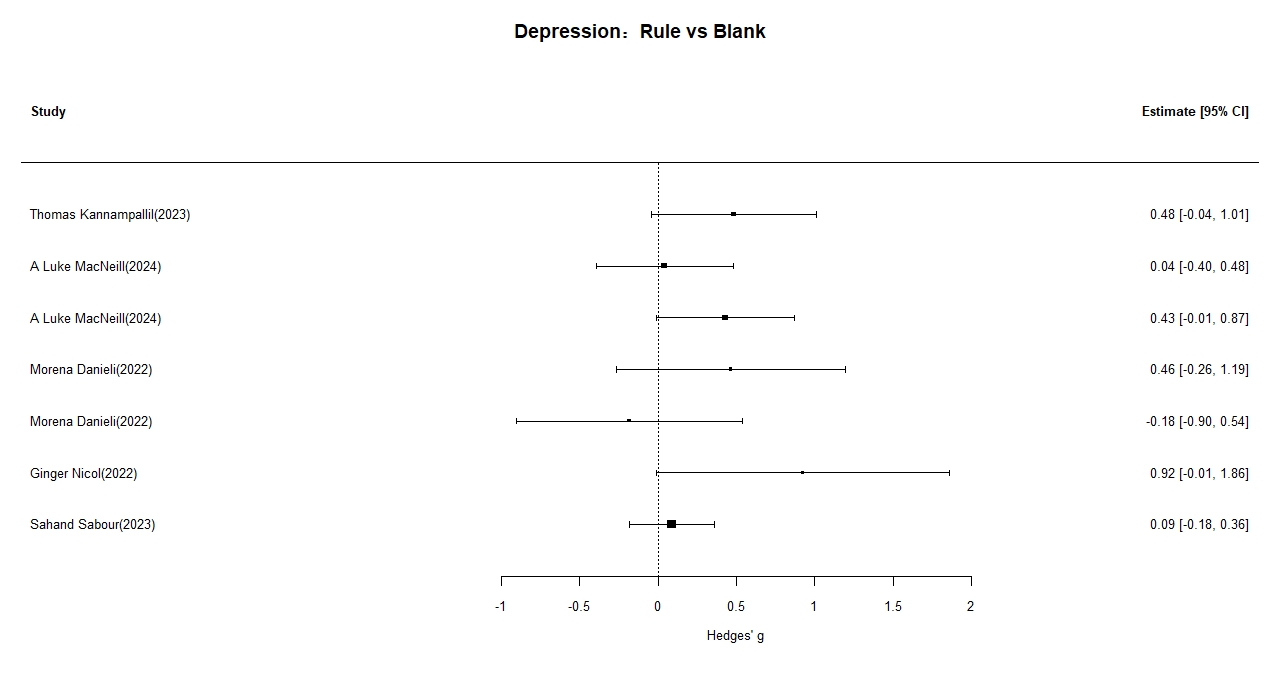


Figure S2. Forest Plot of Depression Interventions - Rule vs Blank


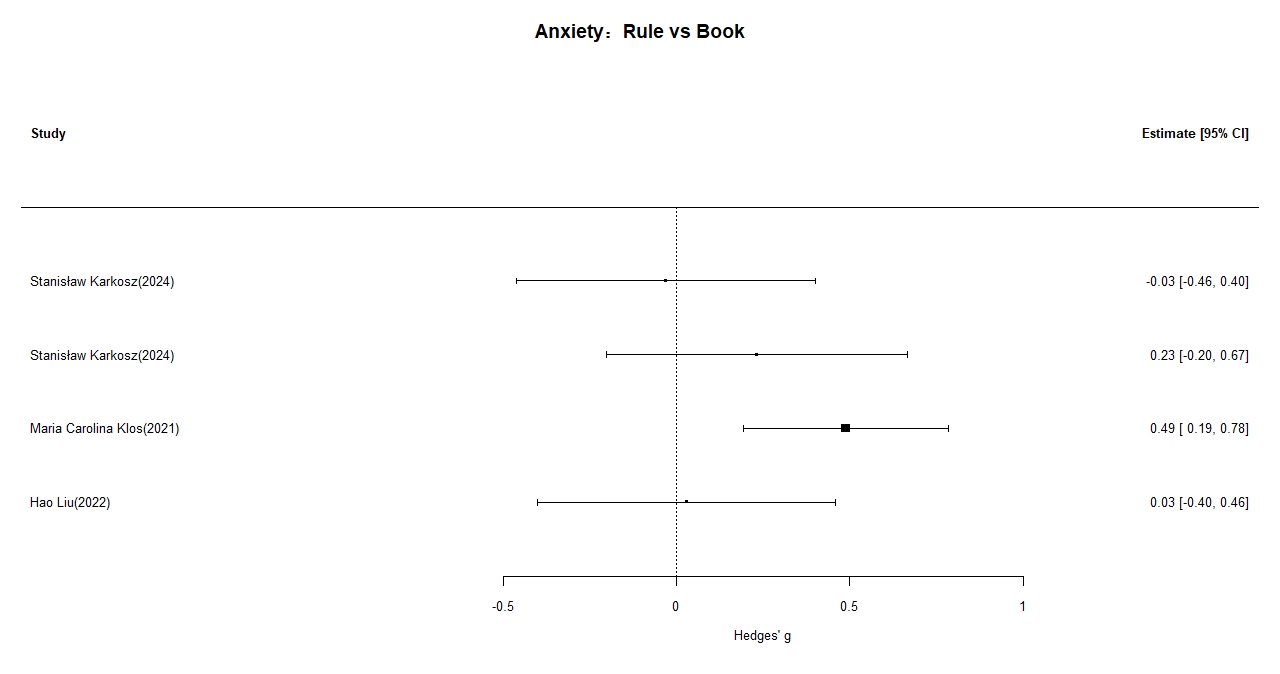


Figure S3. Forest Plot of Anxiety Interventions - Rule vs Book


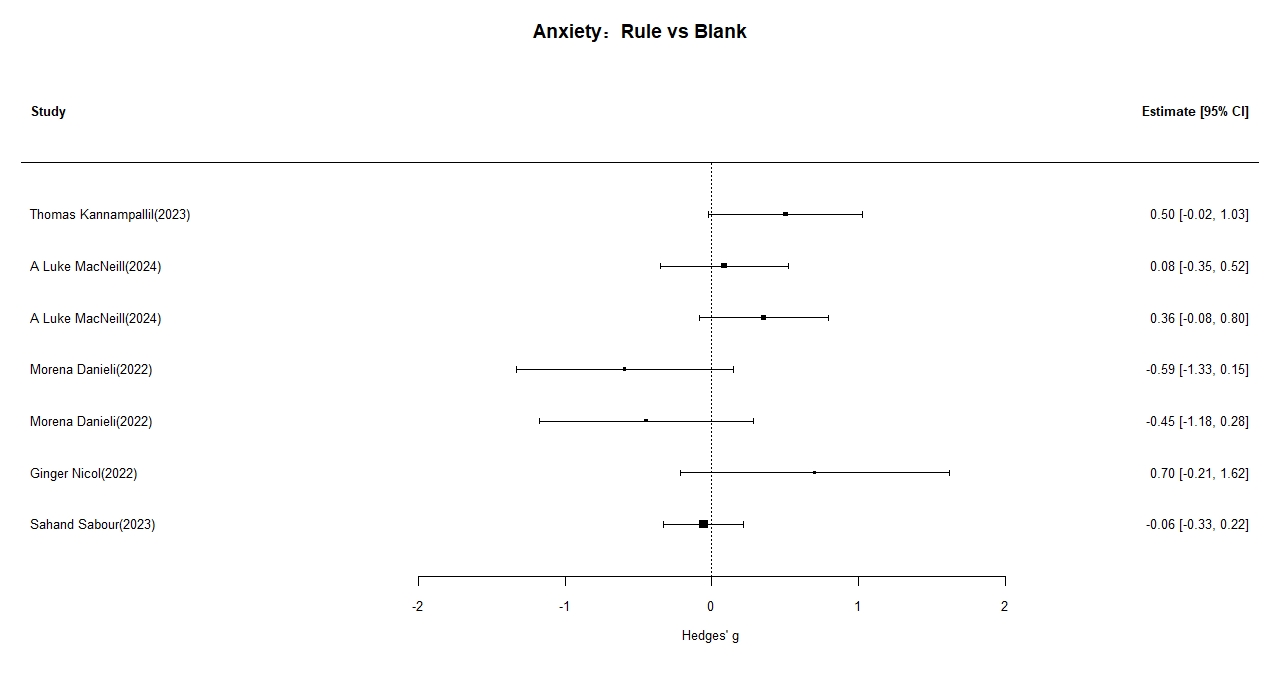


Figure S4. Forest Plot of Anxiety Interventions - Rule vs Blank


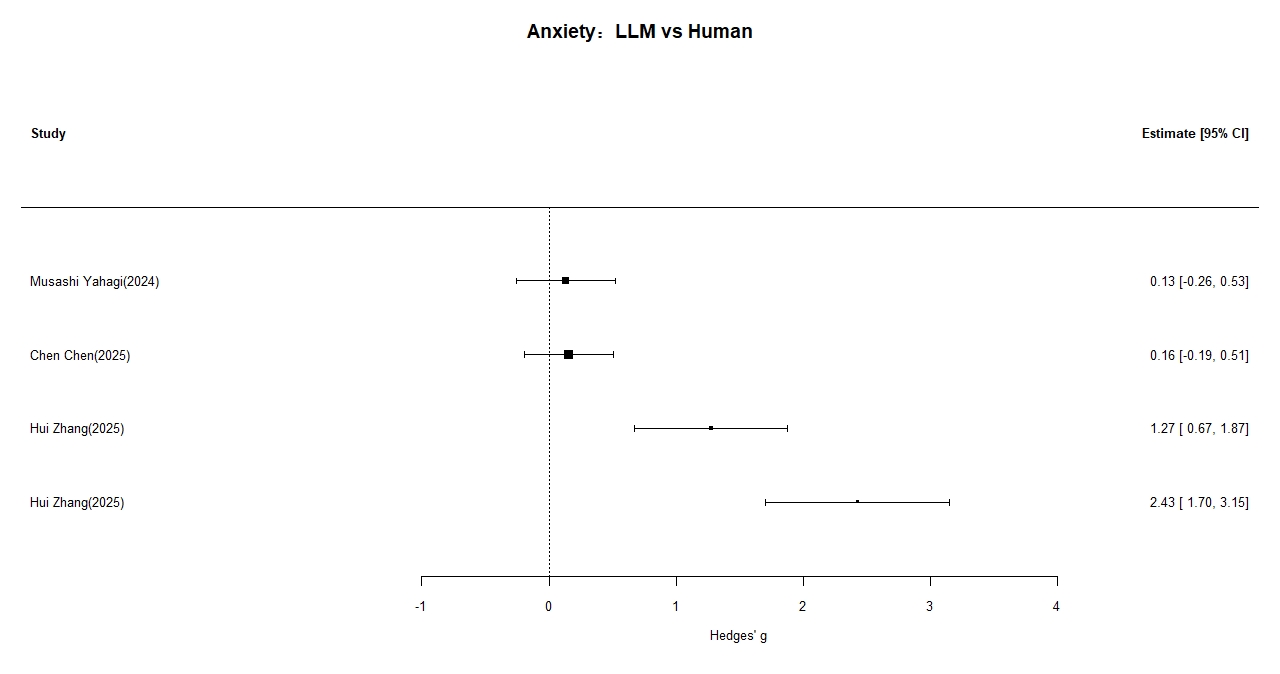


Figure S5. Forest Plot of Anxiety Interventions - LLM vs Human
